# TBC1D15/RAB7-regulated mitochondria-lysosome interaction confers cardioprotection against acute myocardial infarction-induced cardiac injury

**DOI:** 10.7150/thno.46883

**Published:** 2020-09-14

**Authors:** Wenjun Yu, Shiqun Sun, Haixia Xu, Congye Li, Jun Ren, Yingmei Zhang

**Affiliations:** 1 Department of Cardiology and Shanghai Institute of Cardiovascular Diseases, Zhongshan Hospital, Fudan University, Shanghai 200032, China.; 2 Department of Cardiology, Affiliated Hospital of Nantong University, Jiangsu 226001, China.; 3 Department of Cardiology, Xijing Hospital, Air Force Medical University, Xi'an, 710032, China.; 4 Center for Cardiovascular Research and Alternative Medicine, University of Wyoming, Laramie, WY 82071, USA.

**Keywords:** Myocardial infarction, Mitophagy flux, Mitochondria-lysosome contacts, TBC1D15, RAB7

## Abstract

**Rationale:** Ischemic heart disease remains a primary threat to human health, while its precise etiopathogenesis is still unclear. TBC domain family member 15 (TBC1D15) is a RAB7 GTPase-activating protein participating in the regulation of mitochondrial dynamics. This study was designed to explore the role of TBC1D15 in acute myocardial infarction (MI)-induced cardiac injury and the possible mechanism(s) involved.

**Methods:** Mitochondria-lysosome interaction was evaluated using transmission electron microscopy and live cell time-lapse imaging. Mitophagy flux was measured by fluorescence and western blotting. Adult mice were transfected with adenoviral TBC1D15 through intra-myocardium injection prior to a 3-day MI procedure. Cardiac morphology and function were evaluated at the levels of whole-heart, cardiomyocytes, intracellular organelles and cell signaling transduction.

**Results:** Our results revealed downregulated level of TBC1D15, reduced systolic function, overt infarct area and myocardial interstitial fibrosis, elevated cardiomyocyte apoptosis and mitochondrial damage 3 days after MI. Overexpression of TBC1D15 restored cardiac systolic function, alleviated infarct area and myocardial interstitial fibrosis, reduced cardiomyocyte apoptosis and mitochondrial damage although TBC1D15 itself did not exert any myocardial effect in the absence of MI. Further examination revealed that 3-day MI-induced accumulation of damaged mitochondria was associated with blockade of mitochondrial clearance because of enlarged defective lysosomes and subsequent interrupted mitophagy flux, which were attenuated by TBC1D15 overexpression. Mechanistic studies showed that 3-day MI provoked abnormal mitochondria-lysosome contacts, leading to lysosomal enlargement and subsequently disabled lysosomal clearance of damaged mitochondria. TBC1D15 loosened the abnormal mitochondria-lysosome contacts through both the Fis1 binding and the RAB7 GAPase-activating domain of TBC1D15, as TBC1D15-dependent beneficial responses were reversed by interference with either of these two domains both *in vitro* and* in vivo*.

**Conclusions:** Our findings indicated a pivotal role of TBC1D15 in acute MI-induced cardiac anomalies through Fis1/RAB7 regulated mitochondria-lysosome contacts and subsequent lysosome-dependent mitophagy flux activation, which may provide a new target in the clinical treatment of acute MI.

## Introduction

Although much progress has been made in the clinical therapeutics of heart diseases to reduce cardiovascular mortality, ischemic heart disease, especially myocardial infarction (MI), still remains a major threat to human health with somewhat dismal reperfusion effectiveness 1, 2. The course of MI can be clinically staged into hyperacute, acute, subacute and chronic phases 3, 4. Adequate care and treatment during acute phase are critical for the prognosis of patients following MI insult 5, 6. It is thus pertinent to further elucidate the precise pathogenesis of acute MI in an effort to engage effective clinical management regimen. Among plethora of mechanisms postulated for the onset and progression of acute MI, mitochondria have drawn the most attention 2, 7-10. Mitochondria serve as the energy powerhouse for cells with ATP production 8, 11. Upon injury, mitochondria lose their membrane potential and release harmful byproducts of ATP production such as reactive oxygen species (ROS) and pro-apoptotic factors such as cytochrome C, which provoke detrimental effect to cellular function and cell fate 12-14. Indeed, mitochondrial dysfunction has been well consolidated to function as a precursor to cell death 15. Therefore, it is pertinent to maintain stringent mitochondrial quality to control ROS production and promote cell survival.

Mitochondrial quality control involves a dynamic process of fission, fusion, mitophagy, and biogenesis, among which, mitophagy serves as the main safeguard for mitochondrial quality control 16. Mitophagy (selective mitochondrial autophagy) is an important and highly conserved dynamic cytosolic process for removal and recycling of long-lived or damaged mitochondria by lysosomes through PINK1/Parkin- or mitophagy receptors- dependent pathway 14, 17, 18. Although findings from genetically engineered mice have depicted a protective role for mitophagy in various cardiac pathological settings 19-22, a controversy recently emerged for the precise role of autophagy in the setting of cardiac post-infarction 23-25. In particular, autophagic activity is upregulated in ischemic hearts during early stage of infarction at various reported time, while autophagy seems to be compromised during the late stage when all autophagosomes and lysosomes were fused to form autolysosomes. In this context, autolysosomes may accumulate during late stage of acute MI challenge. However, the precise mechanism(s) involved in the accumulation of autolysosomes remains largely unknown.

Autolysosomes accumulation is resulted from blockade of autolysosomes degradation, which may be associated with lysosomal dysfunction 26, 27. In the last few years, functional interactions of intracellular organelles like mitochondria and lysosomes, has drawn the most increasing attention 28. Recent evidence has depicted a critical role for mitochondria-lysosome contacts in the regulation of lysosomal dynamics and maintenance of cellular homeostasis 29. Under normal condition, mitochondria and lysosomes joint together to form mitochondria-lysosome contacts dynamically in healthy cells. The TBC1D15/Fis1/RAB7 signaling cascade is deemed a critical process regulating the mitochondria-lysosome contacts. Active GTP-bound RAB7, a small GTPase from the Rab family, facilitates mitochondria-lysosome contacts and regulates lysosomal transport, fusion and maturation. TBC1D15, a member of the TBC (Tre2/Bub2/Cdc16)-domain-containing protein family, is the GTPase-activating protein (GAP) for RAB7. TBC1D15 regulates RAB7 from an active GTP-bound state into an inactive GDP-bound state upon GTP hydrolysis. TBC1D15 is recruited to mitochondria courtesy of binding with Fis1, an outer mitochondrial membrane protein. Mitochondrial TBC1D15 drives lysosomal RAB7 GTP hydrolysis at mitochondria-lysosome contact sites and then untethers contacts, providing a mechanism for mitochondria to modulate lysosomal dynamics. Although the mitochondria-lysosome contacts are proven to be distinct from damaged mitochondria targeting to lysosomes for degradation, expression of mutant TBC1D15 (Fis1 binding or RAB7-GAP domain, regulating mitochondria-lysosome contacts) may induce abnormally large lysosomes in HeLa cells. This observation denoted the involvement of TBC1D15 in the regulation of lysosome-dependent mitochondrial autophagy under pathological conditions. However, the precise role of TBC1D15 and TBC1D15-regulated mitochondria-lysosome contacts in the heart remains to be elucidated. To this end, this work was designed to determine (i) whether TBC1D15 imposes any effect on cardiac function and mitochondrial quality control following acute MI insult, if any; (ii) whether TBC1D15-induced changes in cardiac structure and function are mediated by alteration in TBC1D15/Fis1/RAB7-mediated mitochondria-lysosome contacts and lysosome-dependent mitophagy.

## Materials and Methods

### Animals and materials

All experimental animal protocols were performed in accordance with the Guide for the Care and Use of Laboratory Animals (NIH Publication No. 85-23, revised 1996 by the US National Institutes of Health) and approved by the Institutional Animal Care and Use Committee of the Zhongshan Hospital Fudan University, Shanghai, China (approval number: 2019-309). Briefly, male C57BL/6J mice (6-8-week-old) were purchased from Shanghai Model Organism Center, Inc. All animals were housed following regular circadian cycle with free access to sterilized water and food.

Dulbecco's Modified Eagle Medium (DMEM), glucose-free DMEM and fetal bovine serum (FBS) were purchased from Gibco (Grand Island, USA). Collagenase Ⅰ was purchased from Worthington (Lakewood, USA). Evans blue and 2,3,5-triphenyltetrazolium chloride (TTC) were obtained from Sigma-Aldrich (St. Louis, USA). *In situ* Cell Death Detection kit (TUNEL) was purchased from Roche (Basel, Switzerland). Reactive Oxygen Species Assay Kit (DCFH-DA), 4-6-diamidino-2-phenylindole (DAPI), RIPA buffer, NP-40 buffer, Enhanced BCA Protein Assay Kit (BCA) and Mitochondrial Membrane Potential Assay Kit (JC-1) were obtained from Beyotime Biotechnology (Shanghai, China). Mito-Tracker Red, Lyso-Tracker Green DND-26, Lyso-Sensor Green DND-189 and ProLong Live Antifade Reagent were purchased from Thermo Fisher Scientific (Waltham, USA). Mitochondria Isolation Kit for Cultured Cells or Tissue (with Dounce Homogenizer) was purchased from Abcam (Cambridge, UK). Adenoviruses encoding mouse Flag-tagged TBC1D15 (wild-type, or the R400K and Δ231-240 mutant) were produced by Obio Technology Corporation (Shanghai, China). Adenoviruses encoding mRFP-GFP-LC3 and mito-Keima were purchased from Hanbio Technology Corporation (Shanghai, China) 30.

### Experimental model

Following 3 days of acclimation, animals were anesthetized with continuously 2% isoflurane inhalation and myocardial infarction (MI) was established as previously described 31. Briefly, to exactly expose the heart, left thoracotomy was performed in the fifth intercostal space. The left anterior descending branch (LAD) of coronary artery was permanently ligated with a 6-0 silk suture. After ligation, the skin was sutured by 4-0 nylon sutures. Mice were carefully placed in a temperature-controlled cage at 37 °C until recovery. To explore the role of TBC1D15, mice were randomized into four groups: Sham-Ad-LacZ, MI-Ad-LacZ, Sham-Ad-TBC1D15 and MI-Ad-TBC1D15 groups. To discern specific domain(s) involved in TBC1D15-offered response in MI, a cohort of sham or MI challenged mice received mutant TBC1D15 (R400K or Δ231-240) adenovirus. Sham-operated mice were subjected to the same procedure except ligation of LAD coronary artery. To achieve cardiac-specific overexpression, the adenovirus-targeted TBC1D15 (wild-type, R400K or Δ231-240 mutant) was directly injected onto intra-myocardium at a dosage of 1.2×10^8^ pfu/ul with a 1:10 dilution using a 29G needle at three distinct sites of the ischemic zone 3 days prior to LAD ligation per the manufacturer's manual 32. Mice transfected with an adenoviral vector encoding β-galactosidase (Ad-LacZ, control viral vector) while receiving sham procedure were used as control group 30. Mice were sacrificed 3 days after MI for further experimentation 33.

### Isolation and culture of primary neonatal mouse cardiomyocytes (NMCMs)

The protocol to isolate primary NMCMs was described previously 34. In brief, neonatal mouse hearts were quickly excised and minced into debris followed by enzymatic digestion with collagenase Ⅰ. After digesting for several times, the digested fragments were placed to sediment for several min, and the cells in supernatants were preplated for 90 min to remove fibroblasts and endothelial cells. Then the residual supernatants with abundant cardiomyocytes were replanted in collagen-coated dishes. NMCMs were cultured in complete DMEM with 4500 mg/L glucose, 4 mM L-glutamine, 110 mg/L sodium pyruvate, 1% (v/v) penicillin/streptomycin and 10% (v/v) FBS, and were then incubated at 95% air, 5% CO_2_ and 37 °C. After incubated for 48 h, the medium was refreshed by complete medium. NMCMs were transfected with adenoviruses encoding LacZ, TBC1D15 (wild-type, R400K or Δ231-240 mutant) and mRFP-GFP-LC3 or mito-Keima at the indicated MOI according to the corresponding instructions.

### Hypoxia protocol of NMCMs

Hypoxia was employed to simulate the MI procedure 35. Briefly, the culture medium of NMCMs was switched to a glucose-free DMEM and cells were incubated in a chamber containing 95% N_2_ and 5% CO_2_ gas mixture (Columbus Instruments) for 9 h before further experimentation. Cells transfected with an adenoviral vector encoding LacZ but not encoding TBC1D15 (wild-type, R400K or Δ231-240 mutant) at the same MOI under normoxic condition were used as the control group. All *in vitro* studies were performed using 3 or 6 independent experiments.

### Cardiac function evaluation

After 3 days of MI, all mice were anesthetized with 2% isoflurane and fixed on a heating pad (37 °C) with a supine position. Cardiac function was evaluated across thoracic region using 2-D guided M-mode echocardiography (VisualSonicsVeVo 2100, Toronto, Canada) equipped with a 15-16 MHz linear transducer. Ejection fraction (EF), fractional shortening (FS), left ventricular end diastolic diameter (LVEDD), left ventricular end systolic diameter (LVESD), left ventricular end diastolic volume (LVEDV), left ventricular end systolic volume (LVESV) and heart rate (HR) were recorded. EF was calculated using the equation of: (LVEDV-LVESV)/LVEDV×100%, whereas FS was calculated using the equation of: (LVEDD-LVESD)/LVEDD×100% 36.

### Measurement of infarct size

After 3 days of MI, mice were anesthetized with 2% isoflurane and injected with 1.5% Evans blue from the aortic root. The hearts were rapidly excised and cut into five 1-mm slices on frozen ice. Then the slices were incubated in 1% 2,3,5-triphenyltetrazolium chloride (TTC) solution for 15 min at 37 ℃ to demarcate the viable and non-viable myocardium. The staining was stopped by ice-cold sterile saline and the slices were fixed in 10% formalin solution. Five consecutive slices from each sample were sequentially imaged using a Canon camera with macro lens. Each slice was weighed. Total left ventricular area, area at risk (AAR) and infarct area from individual slices were measured using the Image J software (National Institute of Health, Version 1.8). The weights of infarct area and AAR from the same slice were calculated as weight of the slice multiplied by the ratio of each area. The overall weights of total left ventricle area, infarct area and AAR from the same heart were summed by five consecutive slices. Ratios of overall AAR/left ventricle area and infarct area/AAR of the heart were calculated. A Pearson coefficient between weight of infarct area and AAR was analyzed and was shown with a ratio parameter (k) 37.

### Determination of interstitial fibrosis

Mouse hearts were arrested in diastole by injection of 10% potassium chloride after anesthesia. Hearts were then rapidly excised and fully fixed in 4% paraformaldehyde at 4 °C. After fixation for at least 24 h, each heart was cut transversely into five consecutive 1-mm slices, embedded in paraffin and sliced into 4 μm thick sections for Masson Trichrome staining. Individual single section was obtained using a light microscope (Leica, Germany). Left ventricular and collagen-positive areas were measured using the Image J software. The ratio of overall interstitial fibrosis/left ventricle from the same heart was calculated by total collagen-positive area normalized to total left ventricle area 32.

### Determination of apoptosis

For study at the cellular or subcellular levels, mice were injected with 1.5% Evans blue from aortic root. Non-ischemic region of the heart showed blue while ischemic region displayed white (necrosis, infarct region) and light red (alive, peri-infarct region) in color. Blue and white tissues were wiped away and the light red tissues about 1 mm away from the border of white tissues were obtained for further study. Apoptosis of myocardium and primary cardiomyocytes were determined using TUNEL staining. After washing with PBS for several times and permeating with 0.3% Triton X-100 for 20 min, tissues or cells were incubated with FITC conjugated dUTP solution for 1 h at 37 °C and were then stained with DAPI for an additional 20 min. Micrographs of TUNEL-positive and DAPI-stained nuclei were captured randomly at 400× magnification using a fluorescence microscope (Leica, Germany) and were counted using Image J. For *in vivo* study, 3 fields per heart, 5 hearts per group were analyzed. For *in vitro* study, 12 fields from 3 independent experiments per group were counted. At least 100 cells per field were counted. The percentage of apoptotic cells was calculated as the ratio of the number of TUNEL-positive cells to that of total DAPI-stained cells 36.

### Transmission electron microscopy (TEM)

Murine heart tissues were fixed in 2% glutaraldehyde for at least 24 h. The tissues were then immersed in 2% osmium tetroxide and 1% aqueous uranyl acetate for 1 h. After washed with a series of ethanol solutions (50%, 70%, 90% and 100%), tissues were transferred to propylene oxide, incubated in a 1:1 mixture of propylene oxide and EMbed 812 (Electron Microscopy Sciences) for 1 h and were then placed in a 70 °C oven to polymerize. Sections (75-80 nm) were cut using an ultramicrotome (Leica, Germany) equipped with a Diatome diamond knife and were collected on 200-mesh copper grids. After poststained in 5% uranyl acetate for 10 min and in Reynold's lead citrate for 5 min, sections were observed using a 40-120 kV transmission electron microscope (Hitachi H600 Electron Microscope, Hitachi, Japan) 36. Ten to twenty microscopic fields from 5 hearts per group were analyzed with at least 1000 mitochondria included per group.

### Determination of mitochondrial membrane potential (MMP)

Primary cardiomyocytes were cultured in disposable confocal dishes. After respective treatments, cells were rinsed with PBS and incubated with 5 μM JC-1 dye at 37 °C for 20 min. Fluorescent cells with a ProLong Live Antifade Reagent were visualized at 630× magnification using a confocal microscope (Leica, Germany). Micrographs of 10 fields from 3 independent experiments per group were counted. In JC-1 assay, the originally green fluorescent dye forms red fluorescent aggregates when they encountered energized mitochondria with higher membrane potential. The fluorescence intensity of red and green was measured using Image J and MMP was calculated as red fluorescence intensity/green fluorescence intensity 36.

### Reactive oxygen species (ROS) detection

For cellular ROS detection, living cardiomyocytes were stained with DCFH-DA fluorescence probe. Cells were rinsed using 37 °C pre-warmed PBS and incubated with 10 μM DCFH-DA (1:1000, no serum) for 20 min at 37 °C. Then, cells were washed with 37 °C DMEM without serum for 3 times and were incubated with a ProLong Live Antifade Reagent. Micrographs of 10 fields from 3 independent experiments per group were obtained at 630× magnification using a Leica confocal microscope. DCFH-DA fluorescence (green) were measured using the Image J software.

### Oxygen consumption rate (OCR)

The oxygen consumption rate (OCR) was analyzed using an XFe96 extracellular flux analyzer (Seahorese Bioscience). Firstly, Seahorse 96-well plates were attached by poly-d-lysine (PDL) (Sigma-Aldrich) for at least 2 h before NMCMs isolation. Then, isolated NMCMs were planted on PDL attached plates at a density of 5×10^5^ cells/well and were analyzed to detect the OCR of NMCMs by sequentially adding the following metabolic regulators including oligomycin A (1 μM), FCCP (1 μM), antimycin A (1 μM) and rotenone (1 μM). OCR was measured using cells from 16 wells per group.

### Mitochondria isolation

Mitochondria were isolated from NMCMs using Abcam's benchtop mitochondria isolation kit. Cells were frozen and thawed to weaken membranes, suspended in Reagent A at 5.0 mg/ml, incubated on ice for 10 min. Then NMCMs were homogenized with a glass Dounce homogenizer, and were then centrifuged at 1000× g for 10 min at 4 °C twice. The supernatant was centrifuged at 12000× g for 10 min at 4 °C, and the remaining pellet was resuspended in Reagent C supplemented with phosphatase/protease inhibitor and frozen at -80 °C. Mitochondrial protein concentration was determined by colorimetry using Enhanced BCA Protein Assay Kit (BCA).

### Immunofluorescence

Murine heart sections and primary cardiomyocytes cultured in confocal dishes were fixed in 4% paraformaldehyde. After washing with PBS for several times and incubating with goat serum containing 0.3% Triton X-100 for 1 h, they were incubated overnight with primary antibody at 4 °C. Then the sections or cells were incubated with the fluorescence-conjugated secondary antibody for 1 h. Cells were further stained with DAPI containing anti-fade reagent. Micrographs (10 fields from 3 independent experiments per group) were obtained using a Leica confocal microscope and fluorescence colocalization (Pearson correlation and Mander coefficient) was analyzed using Image J via the Coloc 2 modality.

### Co-immunoprecipitation and western blot

NMCMs were lysed with NP-40 buffer (50 mM Tris-HCl pH 7.5, 150 mM NaCl, NP-40) containing protease and phosphatase inhibitor cocktails. After centrifugation, the supernatants fractions were subjected to immunoprecipitation with monoclonal IgG and monoclonal FLAG antibodies with protein-A/G beads. The precipitants were analyzed by western blot.

For western blot, protein samples were extracted with a RIPA buffer containing phosphatase and protease inhibitor. Equal quantities of proteins (20 μg) were separated by 10-12% SDS-PAGE, and were then electrophoretic transferred to 0.22 μm PVDF membranes. After blocking with 5% non-fat milk in TBST buffer for 60 min, membranes were incubated with primary antibodies overnight at 4 ˚C. Blots were rinsed three times for 10 min in TBST and incubated with the HRP-conjugated secondary antibody for 1 h at room temperature. Densitometry was detected with the enhanced chemiluminescence (ECL) reagent. All the primary antibodies used for co-immunoprecipitation and western blotting are shown in **Table S1**.

### Real-time polymerase chain reaction (real-time PCR)

Real-time PCR was conducted as described previously 38. Total RNA of cells or heart tissues was harvested using a TRIzol Reagent (Thermo Fisher Scientific, Waltham, USA) and a RNeasy Total RNA Isolation Kit (Qiagen, Hilden, Germany). Isolated RNA was reverse-transcribed using an iScript cDNA Synthesis Kit (Takara BIO, Otsu, Japan). Real-time PCR was performed using iQ SYBR Green Supermix (Bio-Rad Laboratories, Hercules, CA) in the CFX96TM Real-Time System (Bio-Rad Laboratories, Hercules, CA). All primers were purchased from Sangon Biotech Corporation (Shanghai, China) and were presented in **Table S2**. Levels of miR-1 were measured using the mirVana qRT-PCR miRNA Detection Kit (Thermo Fisher Scientific, Waltham, USA) in conjunction with real-time PCR. The mimics and antagomir of miR-1 were both purchased from Thermo Fisher Scientific (Waltham, USA). Mutated nucleotides in the TBC1D15-3'UTR were conducted according to a previous report 39. Luciferase activities were detected using a dual luciferase reporter assay kit (Promega) with a luminometer after 1 g PGL3-target DNA and 0.1 g PRL-TK transfection with lipofectamine 3000 (Thermo Fisher Scientific, Waltham, USA) for 48 h 40.

### Live cell time-lapse imaging

Live cells were imaged using a Leica confocal microscope. For mitochondria-lysosome contacts, cells were incubated with Mito-Tracker Red (mitochondria, red) and Lyso-Tracker Green DND-26 (lysosome, green) for 2 h at the concentrations of 100 nM and 50 nM in combination with a ProLong Live Antifade Reagent for the indicated duration according to the manufacturer's protocol. Images were dynamically recorded at 561-nm for Mito-Tracker Red and 488-nm for Lyso-Tracker Green DND-26. Images were analyzed using the Image J software 29.

### Lysosomal acidification and size

Lysosomal acidification was measured in neonatal cardiomyocytes loaded with 1 mg/ml Lyso-Sensor Green DND-189 for 1 h at 37 °C 41. The Lyso-Sensor dye is a probe that produces green fluorescence in acidic environments and is greener when exposed to more acidic environments. Cells were incubated with a ProLong Live Antifade Reagent to delay fluorescence auto-bleaching. The pictures were captured by a Leica confocal microscope (20-25 fields from 3 independent experiments per group). The fluorescence intensity of Lyso-Sensor Green DND-189 was calculated using the Image J software. The Image J software was used to evaluate the size and number of lysosomes in each cell. The average size of lysosomes was normalized to the number of lysosomes 42, 43.

### Statistical analysis

Data were analyzed with Graph-Pad Prism 8 (GraphPad Software, LLC, San Diego, CA, USA) and expressed as Mean ± SEM. Data distribution was examined using the Shapiro-Wilk normality test. Two groups were compared by Student's *t* test (two-tailed). Multiple groups were compared by one-way ANOVA followed with the Tukey post hoc test. For groups receiving different secondary treatments, two-way ANOVA together with Tukey test were performed to compare the difference. A value of *P <* 0.05 was considered statistically significant.

## Results

### TBC1D15 overexpression partially preserves cardiac function and improves myocardial morphology

**Figure 1:**
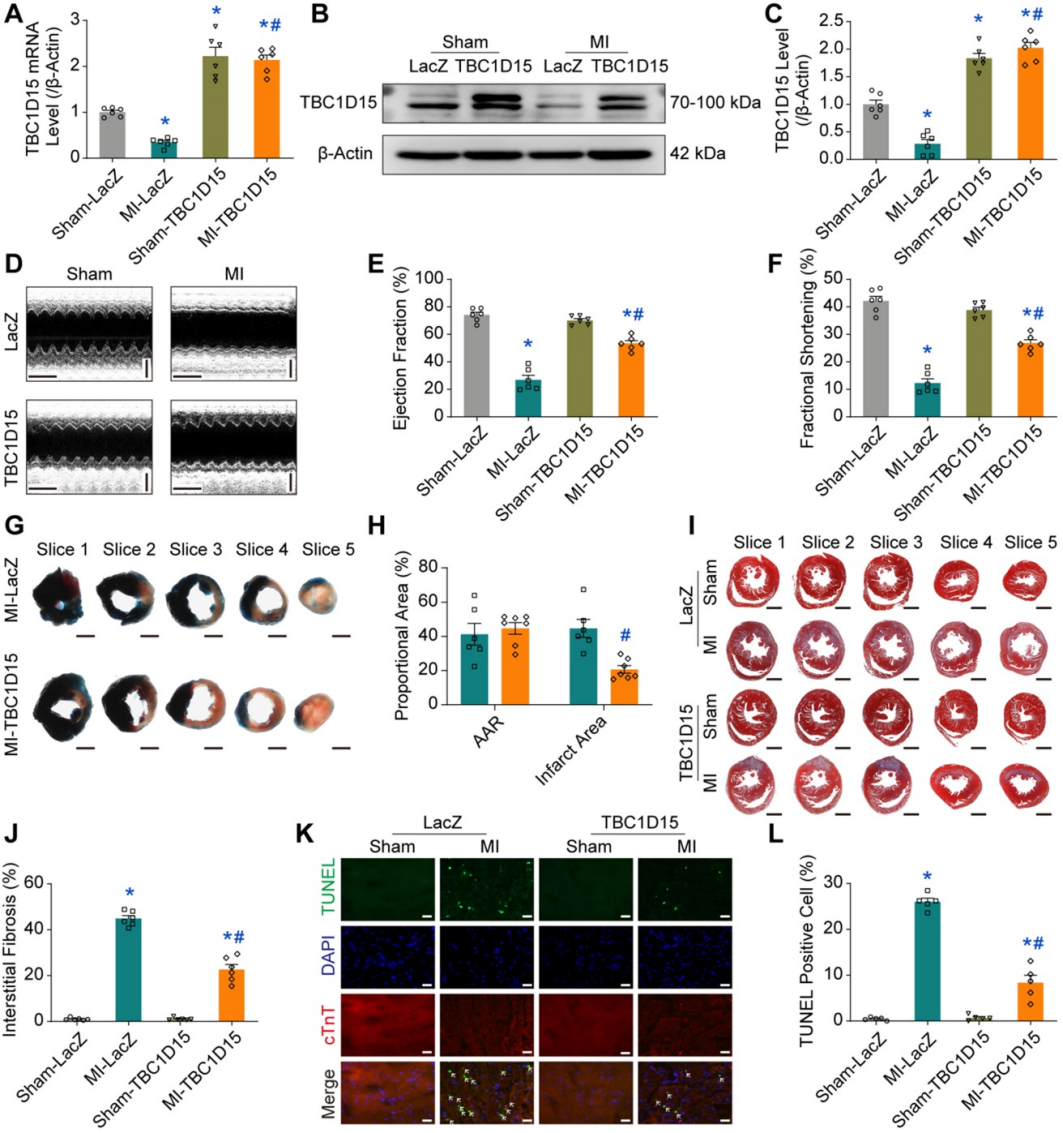
** TBC1D15 overexpression protects against cardiac injury following acute MI. A-C.** Downregulation of TBC1D15 mRNA and protein level (normalized to β-Actin) in peri-infarct myocardium following 3-day MI was reversed by TBC1D15 overexpression (n = 6); **D-F.** Decreases of ejection fraction and fractional shortening following 3-day MI were attenuated by TBC1D15 overexpression (n = 6). Representative echocardiographic images (Scale bar = 200 ms-horizontal and 2 mm-vertical) are shown; **G-H.** Myocardial infarct size evoked by 3-day MI was alleviated by TBC1D15 overexpression (n = 6). Representative five consecutive sections of Evans blue/TTC staining (Scale bar = 1 mm) are displayed. AAR: area at risk; **I-J.** Myocardial interstitial fibrosis evoked by 3-day MI was ameliorated by TBC1D15 overexpression (n = 6). Representative five sections of Masson Trichrome staining (Scale bar = 1 mm) are illustrated; **K-L.** Increase of myocardial apoptosis following 3-day MI was attenuated by TBC1D15 overexpression (n = 5). Representative TUNEL/DAPI/cTnT staining images (Scale bar = 25 µm) are exhibited. The white arrows indicate the TUNEL positive nuclei. Mean ± SEM, * *p* < 0.05 *vs.* Sham-LacZ group; # *p* < 0.05 *vs.* MI-LacZ group.

Our data revealed that both TBC1D15 mRNA (0.346 ± 0.039 *vs.* 1.004 ± 0.040 from Sham, *P* < 0.05, Figure 1A) and protein levels (0.282 ± 0.078 *vs.* 1.000 ± 0.077 from Sham, *P* < 0.05, Figure 1B-C) were overtly downregulated in peri-infarct myocardium 3 days after MI. Since miR-1 was shown to be dramatically upregulated in infarct hearts 40, 44, and loss of miR-1 could be associated with elevation of TBC1D15 levels 39, we deciphered whether TBC1D15 downregulation was due to acute MI-induced miR-1 upregulation. Our results showed that miR-1 level was increased 3 days after MI (3.945 ± 0.363 *vs.* 1.008 ± 0.058 from Sham, *P* < 0.05, Figure S1A). With miR-1 mimics treatment, TBC1D15 mRNA level was reduced in the absence of hypoxia (0.460 ± 0.077 *vs.* 1.008 ± 0.058 from Scramble, *P* < 0.05, Figure S1B). In the presence of TBC1D15-3'UTR mutant treatment, miR-1 mimics failed to induce the reduction described above (Figure S1C). With treatment of the miR-1 antagomir, TBC1D15 mRNA level was increased in the absence (2.141 ± 0.289 *vs.* 1.002 ± 0.031 from Normoxia, *P* < 0.05) or presence of 9 h hypoxia (1.319 ± 0.155 *vs.* 0.210 ± 0.024 from Hypoxia, *P* < 0.05) (Figure S1D). To assess the role of TBC1D15 in acute MI-induced cardiac changes *in vivo*, adenovirus encoding TBC1D15 was delivered to left ventricular myocardium using local myocardial multiple point injection. TBC1D15 overexpression was confirmed 6 days after transfection (3 days after MI). Our result indicated that TBC1D15 adenovirus transfection masked ischemia-induced downregulation of TBC1D15 mRNA (2.137 ± 0.109 *vs.* 0.346 ± 0.039 from MI, *P* < 0.05, Figure 1A) and protein level (2.024 ± 0.098 *vs.* 0.282 ± 0.078 from MI, *P* < 0.05, Figure 1B-C) in peri-infarct myocardium 3 days after MI. Kaplan-Meier survival curve showed that more than 50% mice died within 10 days following MI while this reduced to about 20% by TBC1D15 overexpression (Figure S2B). For further evaluation of the effect of TBC1D15 on acute MI-induced changes in cardiac function, echocardiography was employed to evaluate ejection fraction (EF), fractional shortening (FS), left ventricular end systolic diameter (LVESD), left ventricular end diastolic diameter (LVEDD) and left ventricular end systolic volume (LVESV). As shown in Figure 1D-F, the 3-day MI challenge significantly decreased EF (26.97 ± 3.23 *vs.* 74.10 ± 1.96 from Sham, *P* < 0.05) and FS (12.30 ± 1.57 *vs.* 42.15 ± 1.69 from Sham, *P* < 0.05), the effect of which was overtly attenuated by TBC1D15 overexpression (53.44 ± 2.03 *vs.* 26.97 ± 3.23 from MI, *P* < 0.05 for EF; 26.81 ± 1.21 *vs.* 12.30 ± 1.57 from MI, *P* < 0.05 for FS). Along the same line, LVESD (3.306 ± 0.254 *vs.* 1.964 ± 0.100 from Sham, *P* < 0.05) and LVESV (46.40 ± 8.61 *vs.* 12.44 ± 1.59 from Sham, *P* < 0.05) were dramatically enhanced following MI procedure, the effect of which was partially reversed by TBC1D15 overexpression (2.469 ± 0.158 *vs.* 3.306 ± 0.254 from MI, *P* < 0.05 for LVESD; 22.42 ± 3.77 *vs.* 46.40 ± 8.61 from MI, *P* < 0.05 for LVESV) (Figure S2C-D). TBC1D15 alone exerted no effects on EF, FS, LVESD and LVESV. Neither MI procedure nor TBC1D15 adenovirus, or both, overtly affected LVEDD (Figure S2E). To evaluate the effect of TBC1D15 overexpression on acute MI-induced myocardial morphological changes, Evans blue/TTC staining, Masson Trichrome staining and TUNEL staining were used to evaluate myocardial infarct area, interstitial fibrosis and cardiomyocyte apoptosis, respectively. Our finding revealed that myocardial infarct area (44.73 ± 5.25 *vs.* 0 from Sham), interstitial fibrosis (44.87 ± 1.30 *vs.* 1.04 ± 0.22 from Sham, *P* < 0.05) and cardiomyocyte apoptosis (26.01 ± 0.78 *vs.* 0.48 ± 0.15 from Sham, *P* < 0.05) were significantly upregulated following 3-day MI, the effect of which was relieved by TBC1D15 overexpression (20.66 ± 2.22 *vs.* 44.73 ± 5.25 from MI, *P* < 0.05 for infarct area; 22.69 ± 2.20 *vs.* 44.87 ± 1.30 from MI, *P* < 0.05 for interstitial fibrosis; 8.37 ± 1.62 *vs.* 26.01 ± 0.78 from MI, *P* < 0.05 for apoptosis) (Figure 1G-L and Figure S2F). Consistent with TUNEL staining, apoptotic protein level of Bax was obviously increased (2.388 ± 0.130 *vs.* 1.000 ± 0.095 from Sham, *P* < 0.05) 3 days after MI while anti-apoptotic protein level of Bcl2 level was downregulated (0.359 ± 0.041 *vs.* 1.023 ± 0.071 from Sham, *P* < 0.05). All these changes were partially attenuated by TBC1D15 overexpression (1.588 ± 0.064 *vs.* 2.388 ± 0.130 from MI, *P* < 0.05 for Bax; 0.616 ± 0.044 *vs.* 0.359 ± 0.041 from MI, *P* < 0.05 for Bcl2) (Figure S2G-H). TBC1D15 alone exerted little effects on myocardial infarct area, interstitial fibrosis and cardiomyocyte apoptosis. These findings suggested that TBC1D15 overexpression partially preserved cardiac function and myocardial morphology in the face of acute MI.

### TBC1D15 overexpression attenuates mitochondrial dysfunction

**Figure 2:**
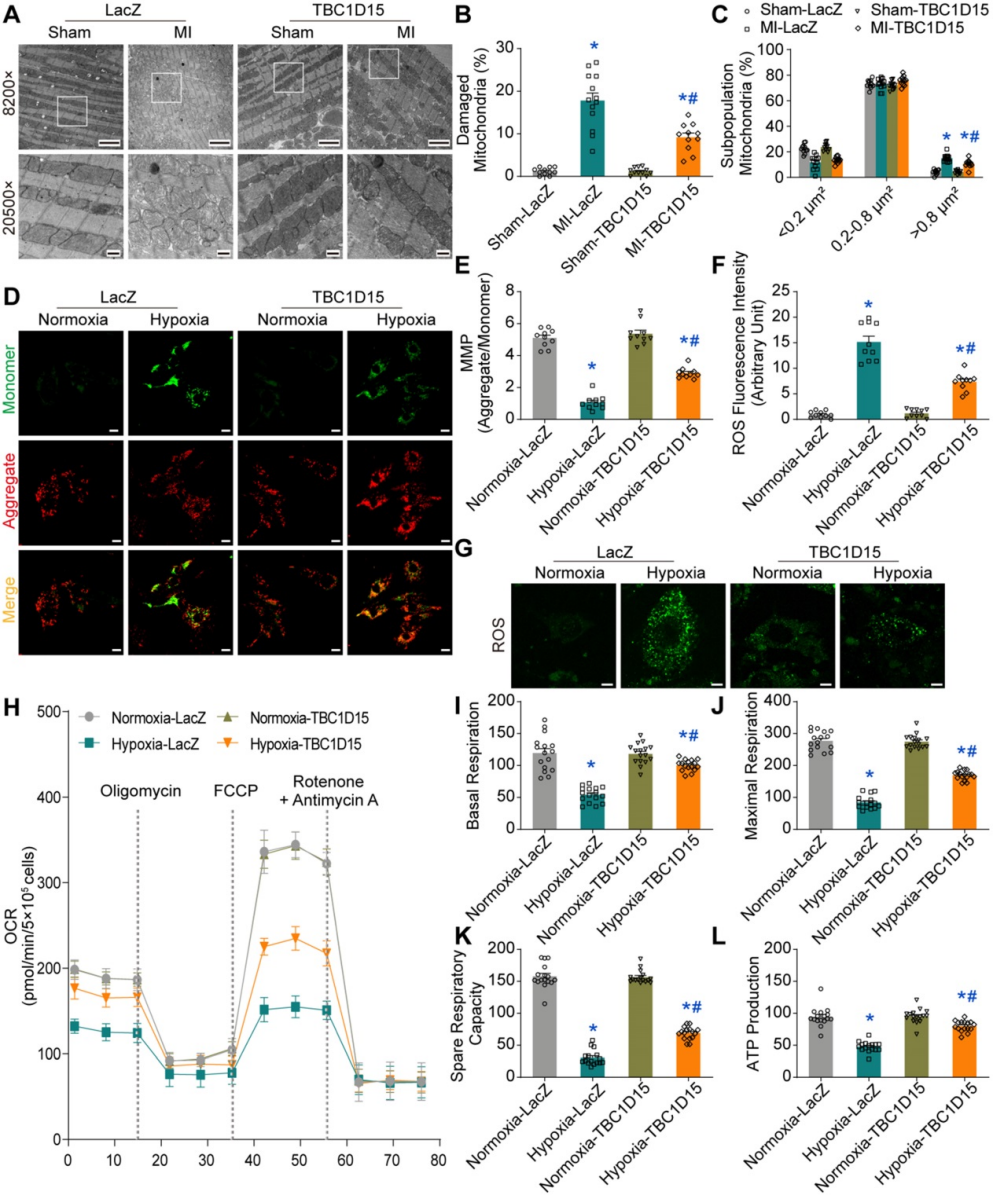
** TBC1D15 alleviates cardiomyocyte mitochondrial dysfunction following acute MI or ischemic stress. A-C.** Increases of damaged mitochondria and mitochondrial size following 3-day MI were attenuated by TBC1D15 overexpression (n = 11-13). Representative TEM images of mitochondrial morphology (Scale bar = 2 µm-upper and 500 nm-lower) are shown. Rectangles are magnified views. Mean ± SEM, * p < 0.05 *vs.* Sham-LacZ group; # *p* < 0.05 *vs.* MI-LacZ group;** D-E.** Mitochondrial membrane potential (MMP) loss following 9-h hypoxia was alleviated by TBC1D15 overexpression (n = 10). Representative images of JC-1 staining (Scale bar = 10 µm) are displayed; **F-G.** Reactive oxygen species (ROS) accumulation following 9-h hypoxia was ameliorated by TBC1D15 overexpression (n = 10). Representative images of DCFH-DA staining (Scale bar = 10 µm) are exhibited; **H-L.** Decreases of basal respiration, maximal respiration, spare respiratory capacity and ATP production following 9-h hypoxia were attenuated by TBC1D15 overexpression (n = 16). Oxygen consumption rate (OCR) curves are shown. Mean ± SEM, * *p* < 0.05 *vs.* Normoxia-LacZ group; # *p* < 0.05 *vs.* Hypoxia-LacZ group.

Given that mitochondria are essential for cardiomyocyte function, we next focused on TBC1D15-dependent responses in mitochondrial function using TEM, JC-1 staining, ROS staining and OCR. TEM results exhibited elevated percentage of damaged mitochondria (characterized by swelling, deformation, cristae fracture and rupture) in cardiomyocytes following 3-day MI (17.80 ± 1.78 *vs.* 1.22 ± 0.25 from Sham, *P* < 0.05), an effect that was partially reversed by TBC1D15 overexpression (9.19 ± 1.00 *vs.* 17.80 ± 1.78 from MI, *P* < 0.05) (Figure 2A-B). Data from sham group showed that area of more than 1000 mitochondria can be categorized into 3 categories, < 0.2 μm^2^, 0.2-0.8 μm^2^ and > 0.8 μm^2^. Our results revealed a significant rise in the percentage of mitochondria > 0.8 μm^2^ (15.03 ± 0.77 *vs.* 4.29 ± 0.59 from Sham, *P* < 0.05) and overtly decreased proportion in mitochondria < 0.2 μm^2^ (11.68 ± 1.45 *vs.* 22.50 ± 1.06 from Sham, *P* < 0.05) from MI challenged hearts, indicating more swollen mitochondria. TBC1D15 overexpression significantly decreased the percentage of mitochondria > 0.8 μm^2^ (10.87 ± 0.95 *vs.* 15.03 ± 0.77 from MI, *P* < 0.05) with no effect on the proportion of mitochondria < 0.2 μm^2^. Interestingly, proportion of mitochondria between 0.2-0.8 μm^2^ was comparable among all groups (Figure 2C). Although the number of total mitochondria was decreased 3 days after MI (101.8 ± 3.4 *vs.* 129.2 ± 7.6 from Sham, *P* < 0.05), TBC1D15 overexpression exerted no influence on the decrease of mitochondria (Figure S3A), consistent with the changes of PGC1α protein level (Figure S3B-C), a critical molecule involved in mitochondrial biogenesis. Mitochondria in adult cardiomyocytes can be categorized into 3 distinct populations, peri-nuclear, interfibrillar, and subsarcolemmal mitochondria. Our data showed little difference in the proportion distribution of 3 distinct mitochondrial populations in all groups (Figure S3D). JC-1 aggregate (red)/monomer (green) ratio is an indicator of mitochondrial membrane potential (MMP). Our results showed that MMP was reduced in response to 9-h hypoxia (1.064 ± 0.141 *vs.* 5.104 ± 0.176 from Normoxia, *P* < 0.05) although such phenomenon was partially reversed by TBC1D15 overexpression (2.901 ± 0.107 *vs.* 1.064 ± 0.141 from Hypoxia, *P* < 0.05) (Figure 2D-E). In addition, ROS (green) was dramatically accumulated in response to 9-h hypoxia (15.15 ± 1.16 *vs.* 0.96 ± 0.21 from Normoxia, *P* < 0.05), an effect that was mitigated by TBC1D15 (7.35 ± 0.54 *vs.* 15.15 ± 1.16 from Hypoxia, *P* < 0.05) (Figure 2F-G). Oxygen consumption rate (OCR) detection revealed that basal respiration (54.3 ± 3.0 *vs.* 119.4 ± 6.9 from Normoxia, *P* < 0.05), maximal respiration (84.9 ± 5.0 *vs.* 277.2 ± 7.4 from Normoxia, *P* < 0.05), spare respiratory capacity (30.6 ± 3.0 *vs.* 157.8 ± 4.6 from Normoxia, *P* < 0.05) and ATP production (47.7 ± 2.0 *vs.* 94.3 ± 3.8 from Normoxia, *P* < 0.05) were all suppressed 9 h after hypoxia, the effects of which were partially reversed by TBC1D15 (100.4 ± 2.2 *vs.* 54.3 ± 3.0 from Hypoxia, *P* < 0.05 for basal respiration; 169.3 ± 3.6 *vs.* 84.9 ± 5.0 from Hypoxia, *P* < 0.05 for maximal respiration; 68.9 ± 2.5 *vs.* 30.6 ± 3.0 from Hypoxia, *P* < 0.05 for spare respiratory capacity; 79.9 ± 2.1 *vs.* 47.7 ± 2.0 from Hypoxia, *P* < 0.05 for ATP production) (Figure 2H-L). TBC1D15 alone exerted no discernable responses in ultrastructure, MMP, ROS production and OCR indices. These findings suggested that TBC1D15 overexpression attenuated mitochondrial dysfunction following acute MI or long-term hypoxia.

### TBC1D15 overexpression improves autophagy and mitophagy flux

**Figure 3:**
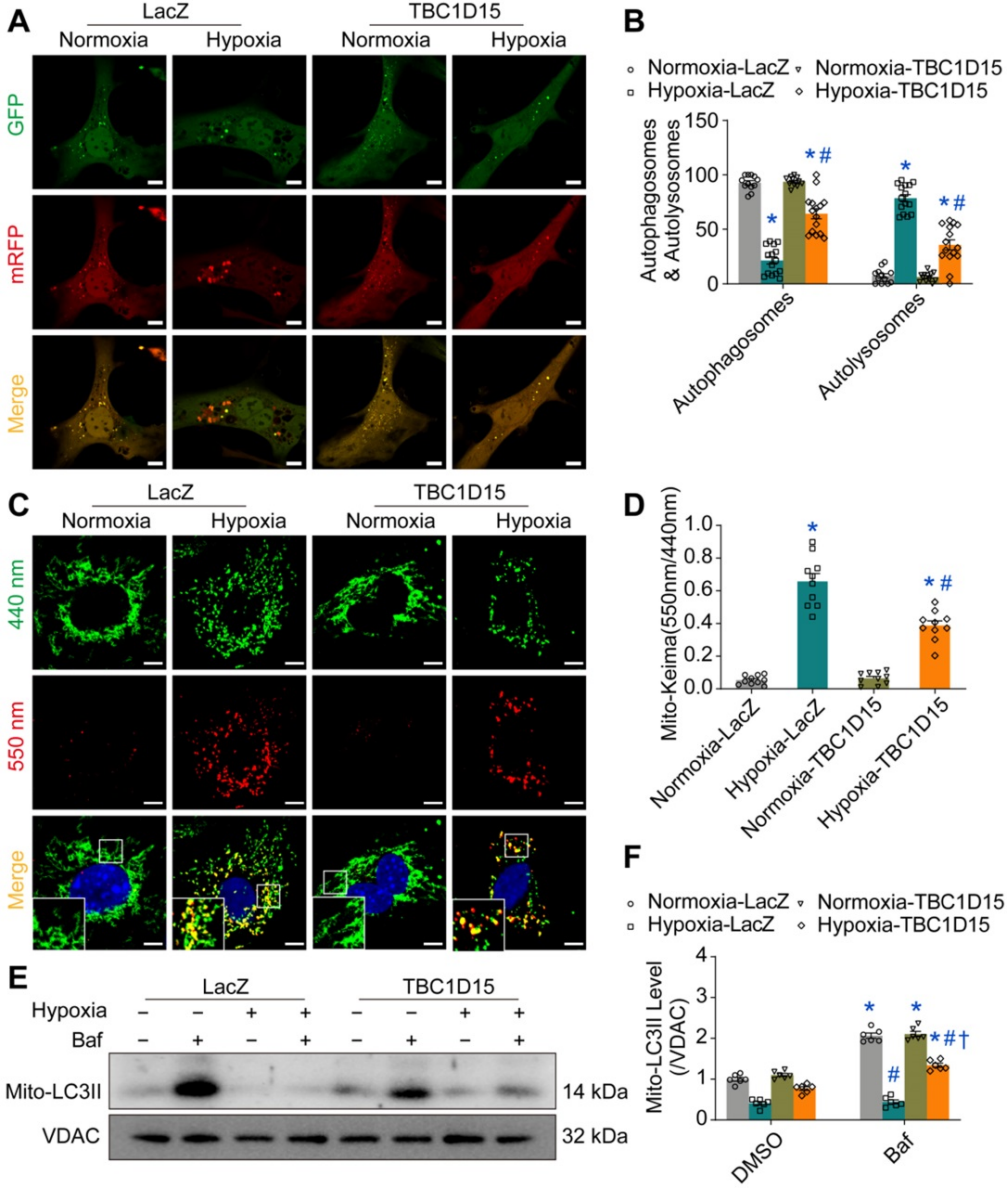
** TBC1D15 activates cardiomyocyte mitophagy flux suppressed by hypoxia. A-B.** Decrease of autophagosomes and increase of autolysosomes following 9-h hypoxia were attenuated by TBC1D15 overexpression (n = 12-15). Representative images of mRFP-GFP-LC3 transfection (Scale bar = 10 µm) are shown; **C-D.** Increase of mito-Keima fluorescence intensity ratio (550 nm/440 nm) following 9-h hypoxia was alleviated by TBC1D15 overexpression (n = 10). Representative images of mito-Keima transfection (Scale bar = 5 µm) are displayed. Rectangles denote magnified views. Mean ± SEM, * *p* < 0.05 *vs.* Normoxia-LacZ group; # *p* < 0.05 *vs.* Hypoxia-LacZ group; **E-F.** Hypoxia (9 h)-induced suppression of mito-LC3II elevation (normalized to VDAC) in response to bafilomycin A1 (Baf) was ameliorated by TBC1D15 overexpression in NMCMs (n = 6). Bafilomycin A1 was administrated at 100 nM for 2 h. Mean ± SEM, * *p* < 0.05 *vs.* corresponding group without Bafilomycin A1; # *p* < 0.05 *vs.* Normoxia-LacZ-Baf group; † *p* < 0.05 *vs.* Hypoxia-LacZ-Baf group.

As damaged mitochondria prompt ROS production and cell death, it is plausible to speculate that the TBC1D15-improved mitochondrial integrity may be due to facilitated mitochondrial autophagy clearance. We next examined autophagy activity in cardiomyocytes following MI. Our results depicted that LC3II was upregulated (1.642 ± 0.063 *vs.* 1.000 ± 0.048 from Sham, *P* < 0.05) while p62 level was downregulated (0.422 ± 0.041 *vs.* 1.000 ± 0.068 from Sham, *P* < 0.05) 2 h following MI. However, LC3II level was decreased (0.514 ± 0.091 *vs.* 1.000 ± 0.048 from Sham, *P* < 0.05) while p62 was upregulated (1.873 ± 0.161 *vs.* 1.000 ± 0.068 from Sham, *P* < 0.05) 72 h following MI (Figure S4A-C). This finding suggested that autophagy flux was enhanced during early acute phase of MI although it dropped accompanied by poor autophagosome formation during late acute phase of MI. Interestingly, interrupted autophagy activity induced by 72-h MI was partially reversed by TBC1D15 (0.711 ± 0.032 *vs.* 0.387 ± 0.025 from MI, *P* < 0.05 for LC3II; 2.164 ± 0.200 *vs.* 3.651 ± 0.268 from MI, *P* < 0.05 for p62) (Figure S4D-F). In line with the LC3II finding, TEM imaging showed that the 72-h MI procedure reduced autophagosome (a double membrane structure containing long-lived proteins or damaged organelles) number (0.692 ± 0.175 *vs.* 4.182 ± 0.444 from Sham, *P* < 0.05), the effect of which was mitigated by TBC1D15 (2.182 ± 0.352 *vs.* 0.692 ± 0.175 from MI, *P* < 0.05) (Figure S4G-H). For mitochondrial autophagy detection, our data noted downregulated mitochondrial LC3II (0.355 ± 0.028 *vs.* 1.000 ± 0.045 from Sham, *P* < 0.05) while upregulated mitochondrial p62 (2.582 ± 0.143 *vs.* 1.000 ± 0.055 from Sham, *P* < 0.05) together with elevated Tom40 (an outer mitochondrial membrane protein) (3.551 ± 0.210 *vs.* 1.000 ± 0.074 from Sham, *P* < 0.05) and Tim23 (an inner mitochondrial membrane protein) (2.472 ± 0.087 *vs.* 1.000 ± 0.088 from Sham, *P* < 0.05) levels following 72-h MI, the effects of which were alleviated by TBC1D15 overexpression (0.634 ± 0.030 *vs.* 0.355 ± 0.028 from MI, *P* < 0.05 for LC3II; 1.541 ± 0.089 *vs.* 2.582 ± 0.143 from MI, *P* < 0.05 for p62; 2.232 ± 0.105 *vs.* 3.551 ± 0.210 from MI, *P* < 0.05 for Tom40; 1.830 ± 0.117 *vs.* 2.472 ± 0.087 from MI, *P* < 0.05 for Tim23) (Figure S5A-E). Immunofluorescent data further revealed that 9-h hypoxia suppressed COXIV (green, mitochondria) and LC3 (red, isolated membrane, initiation of autophagy) colocation (0.055 ± 0.008 *vs.* 0.221 ± 0.021 from Normoxia, *P* < 0.05 for Pearson; 0.391 ± 0.041 *vs.* 0.733 ± 0.042 from Normoxia, *P* < 0.05 for Mander), an indicator of mitochondrial autophagosome, which was enhanced by TBC1D15 overexpression (0.681 ± 0.061 *vs.* 0.055 ± 0.008 from Hypoxia, *P* < 0.05 for Pearson; 0.904 ± 0.034 *vs.* 0.391 ± 0.041 from Hypoxia, *P* < 0.05 for Mander) (Figure S5F-H). These findings suggested that acute MI or long-term hypoxia induced interrupted mitochondrial autophagy activity, which were also mitigated by TBC1D15. To further detect autophagy or mitophagy flux, mRFP-GFP-LC3 and mito-Keima adenoviruses transfection were conducted. For unstressed cells, mRFP-GFP-LC3 shows yellow fluorescence while mito-Keima manifests green fluorescence. When autophagosomes fuse with acidified lysosomes to form autolysosomes, mRFP-GFP-LC3 shows red fluorescence while mito-Keima manifests red fluorescence. Our results showed that overt accumulation of mRFP-LC3 (red, autolysosomes) (78.55 ± 3.28 *vs.* 7.30 ± 1.96 from Normoxia, *P* < 0.05) along with decreased mRFP-GFP-LC3 (yellow, autophagosomes) (21.45 ± 3.28 *vs.* 92.70 ± 1.96 from Normoxia, *P* < 0.05) was noted in 9-h hypoxic cardiomyocytes, the effect of which was alleviated by TBC1D15 (35.68 ± 4.65 *vs.* 78.55 ± 3.28 from Hypoxia, *P* < 0.05 for mRFP-LC3; 64.32 ± 4.65 *vs.* 21.45 ± 3.28 from Hypoxia, *P* < 0.05 for mRFP-GFP-LC3) (Figure 3A-B). Meanwhile, mito-Keima also showed that Keima dots (red, autolysosomes) were accumulated in 9-h hypoxic cardiomyocytes (0.657 ± 0.048 *vs.* 0.054 ± 0.009 from Normoxia, *P* < 0.05), which was also mitigated by TBC1D15 (0.388 ± 0.029 *vs.* 0.657 ± 0.048 from Hypoxia, *P* < 0.05) (Figure 3C-D). Western blot analysis indicated that mitochondrial LC3II was dramatically increased in response to bafilomycin A1 (Baf) (2.065 ± 0.065 *vs.* 1.000 ± 0.047 from Normoxia, *P* < 0.05), a lysosomal V-ATPase inhibitor blocking autophagy flux, under normal environment. Upon 9-h hypoxia challenge, bafilomycin A1 failed to induce mitochondrial LC3II rises although such effect was nullified with TBC1D15 overexpression (1.342 ± 0.049 *vs.* 0.770 ± 0.044 from Hypoxia-TBC1D15, *P* < 0.05) (Figure 3E-F). These suggested that TBC1D15 overexpression improved autophagy and mitophagy flux which were suppressed following acute MI or long-term hypoxia.

### Autophagy flux inhibition abolishes TBC1D15-dependent cardioprotective effects

**Figure 4:**
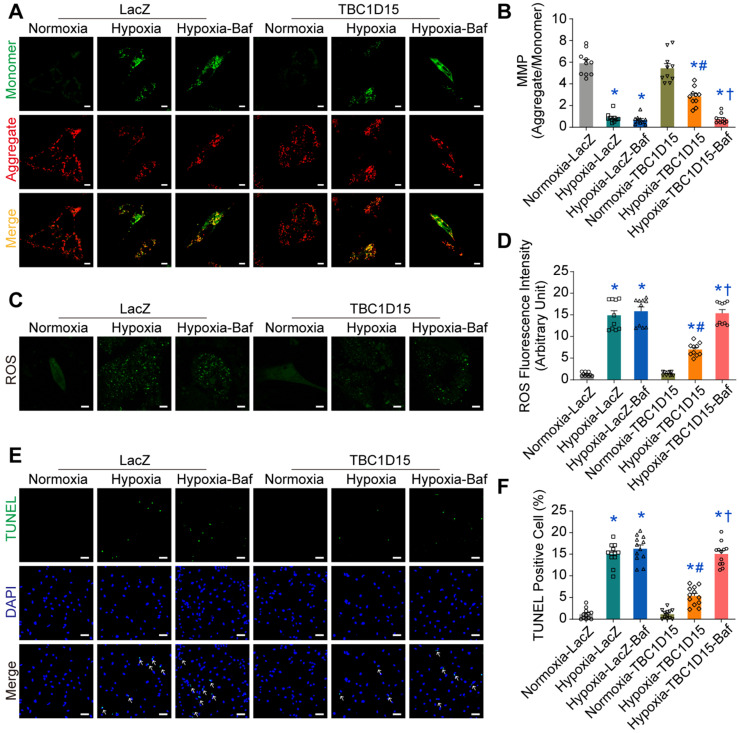
** Inhibition of autophagy flux abolishes TBC1D15 protective effects on cardiomyocytes.** NMCMs were transfected with LacZ or TBC1D15 adenovirus in the absence or presence of 9-h hypoxia. Bafilomycin A1 (Baf) was administrated at 100 nM for 2 h. **A-B.** Hypoxia-induced mitochondrial membrane potential (MMP) loss was attenuated by TBC1D15 overexpression, the effect of which was canceled by Baf (n = 10). Representative images of JC-1 staining (Scale bar = 10 µm) are shown; **C-D.** Hypoxia-induced cardiomyocyte reactive oxygen species (ROS) accumulation was alleviated by TBC1D15 overexpression, the effect of which was abolished by Baf (n = 10). Representative images of DCFH-DA staining (Scale bar = 10 µm) are displayed; **E-F.** Hypoxia-induced cardiomyocyte apoptosis was ameliorated by TBC1D15 overexpression, the effect of which was nullified by Baf (n = 12). Representative images of TUNEL/DAPI staining (Scale bar = 25 µm) are exhibited. The white arrows indicate TUNEL positive nuclei. Mean ± SEM, * *p* < 0.05 *vs.* Normoxia-LacZ group; # *p* < 0.05 *vs.* Hypoxia-LacZ group; † *p* < 0.05 *vs.* Hypoxia-TBC1D15 group.

To further explore the role of autophagy or mitophagy flux in TBC1D15-dependent responses in long-term hypoxic cardiomyocytes, autophagy flux inhibitor bafilomycin A1 (Baf) was used. Then, JC-1, ROS and TUNEL staining were performed. Our data revealed that 9-h hypoxia decreased MMP (0.863 ± 0.137 *vs.* 5.907 ± 0.357 from Normoxia, *P* < 0.05), an effect that was nullified by TBC1D15 overexpression (2.831 ± 0.271 *vs.* 0.863 ± 0.137 from Hypoxia, *P* < 0.05) (Figure 4A-B). Meanwhile, 9-h hypoxia overtly increased ROS production (14.88 ± 1.06 *vs.* 1.30 ± 0.14 from Normoxia, *P* < 0.05), the effect of which was attenuated by TBC1D15 overexpression (6.99 ± 0.47 *vs.* 14.88 ± 1.06 from Hypoxia, *P* < 0.05) (Figure 4C-D). Furthermore, 9-h hypoxia obviously increased TUNEL-positive cells (15.02 ± 0.72 *vs.* 1.15 ± 0.35 from Normoxia, *P* < 0.05), which was attenuated by TBC1D15 overexpression (5.36 ± 0.61 *vs.* 15.02 ± 0.72 from Hypoxia, *P* < 0.05) (Figure 4E-F). Inhibition of autophagy flux using bafilomycin A1 mitigated TBC1D15-dependent cardioprotection with no more detrimental effects on 9-h hypoxia-induced cardiomyocytes abnormalities (0.747 ± 0.139 *vs.* 2.831 ± 0.271 from Hypoxia-TBC1D15, *P* < 0.05 for MMP; 15.37 ± 0.83 *vs.* 6.99 ± 0.47 from Hypoxia-TBC1D15, *P* < 0.05 for ROS; 15.07 ± 0.77 *vs.* 5.36 ± 0.61 from Hypoxia-TBC1D15, *P* < 0.05 for TUNEL). These findings suggested that autophagy flux regulation was involved in TBC1D15-dependent cardio-protective effects.

### Fis1 binding and RAB7-GAP domains are involved in TBC1D15-dependent lysosomal acidification restoration and subsequent mitophagy regulation

**Figure 5:**
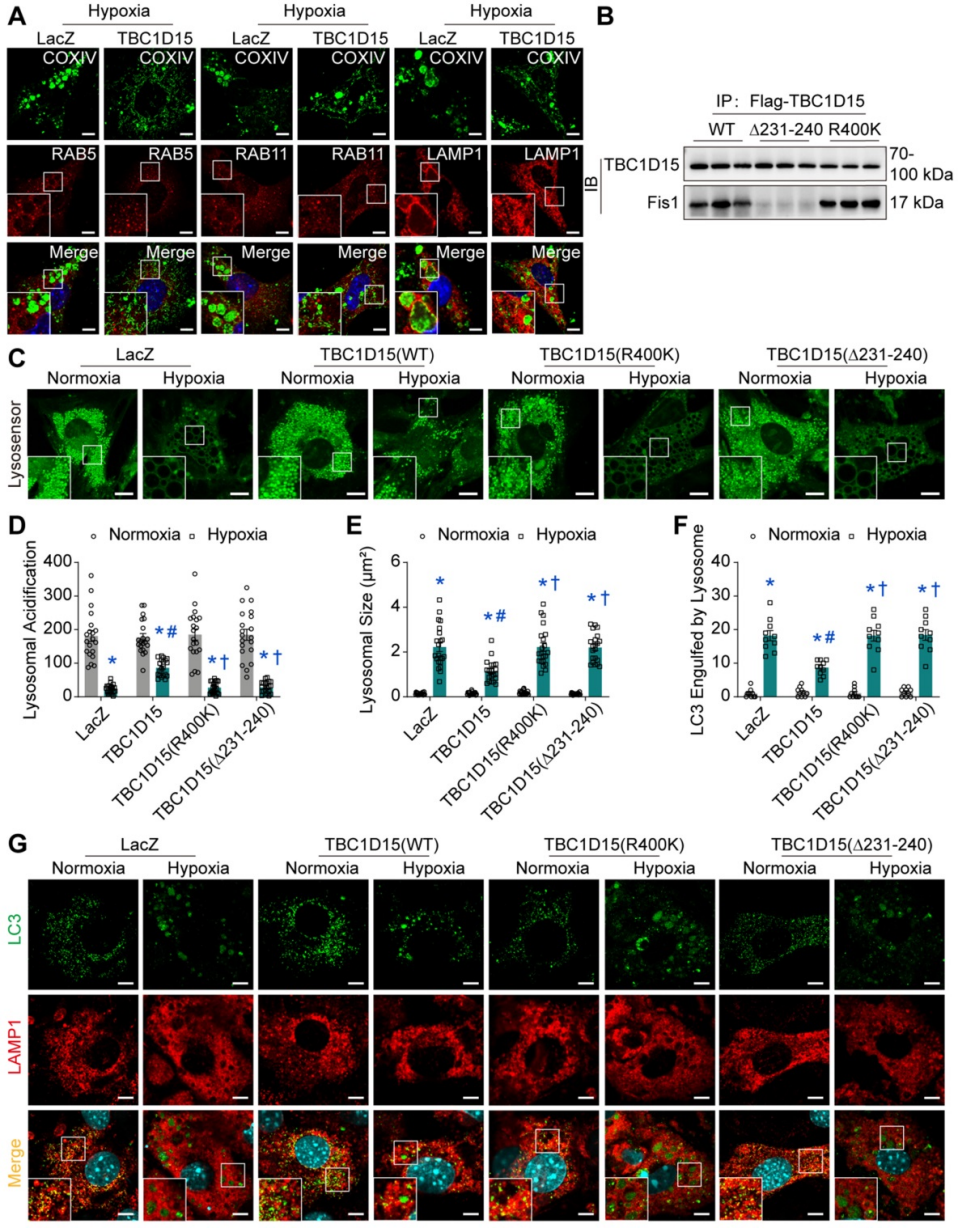
** TBC1D15 overexpression restores lysosomal size and acidic degradation dependent on its Fis1 binding and RAB7-GAP domains.** NMCMs were transfected with LacZ or TBC1D15 adenoviruses (WT, R400K or Δ231-240) in the absence or presence of 9-h hypoxia. **A.** TBC1D15 attenuated hypoxia-induced engulfment of COXIV positive “massive aggregates” (mitochondria, green) by LAMP1 (late lysosomes, red), but not by RAB5 (early lysosomes, red) or RAB11 (recycling lysosomes, red). Representative immunofluorescence images of COXIV together with RAB5, RAB11 and LAMP1 (Scale bar = 5 µm) are shown. Rectangles are magnified views; **B.** TBC1D15 bound with Fis1 in NMCMs transfected with WT and R400K mutant adenoviruses, but not in Δ231-240 mutant transfected cells; **C-E.** Hypoxia-induced decrease of lysosomal acidification and increase of lysosomal size were ameliorated by TBC1D15 overexpression, but not by mutant TBC1D15 (R400K) or TBC1D15 (Δ231-240) (n = 20-22). Representative images of Lyso-Sensor Green DND-189 staining (Scale bar = 7.5 µm) are displayed. Rectangles denote magnified views; **F-G.** Hypoxia-induced engulfment of LC3-positive “granules” (green) by LAMP1 (red) was alleviated by TBC1D15 overexpression, but not by mutant TBC1D15 (R400K) or TBC1D15 (Δ231-240) (n = 10). Representative immunofluorescence images of LAMP1 and LC3 (Scale bar = 5 µm) are exhibited. Rectangles exhibit magnified views. Mean ± SEM, * *p* < 0.05 *vs.* corresponding Normoxia group; # *p* < 0.05 *vs.* Hypoxia-LacZ group; † *p* < 0.05 *vs.* Hypoxia-TBC1D15 group.

Given the decreased autophagosome formation in hypoxic cardiomyocytes, it is plausible to speculate that autolysosomes accumulation could be due to the blockade of autolysosome degradation, which may be associated with endosomal or lysosomal dysfunction. We went on to examine endosomal and lysosomal function following long-term hypoxia in cardiomyocytes. Firstly, immunofluorescence was performed to discriminate endosomes and lysosomes using COXIV (green, mitochondria) and RAB5 (red, early endosomes) 45, RAB11 (red, recycling endosomes) 46 and LAMP1 (red, lysosomes) 47. Under 9-h hypoxia, enlarged vacuoles (red) were observed and positive with LAMP1, not with RAB5 or RAB11, indicative of enlargement of lysosomes, suggesting that lysosomal dysfunction may be involved in mitophagy flux suppression following long-term hypoxia (Figure 5A). Furthermore, the COXIV positive “massive aggregates” (green) were also observed and encompassed by LAMP1 positive enlarged vacuoles (red), not by RAB5 or RAB11, the effect of which was improved by TBC1D15 (Figure 5A). This suggested that damaged mitochondria (COXIV) encompassed by enlarged lysosomes (LAMP1) were unable to be digested by dysfunctional lysosomes. Next, we examined lysosomal function in cardiomyocytes following a 9-h hypoxia procedure. TBC1D15, a RAB7-GAP recruited to mitochondria by the mitochondrial protein Fis1 to facilitate RAB7 GTP hydrolysis, contains two distinct functional domains (Fis1 binding and RAB7-GAP domains) 29. To discern the role of these two domains in TBC1D15-regulated cardiac benefit against ischemia, two mutants of TBC1D15 domains (R400K and Δ231-240 mutants for RAB7-GAP and Fis1 binding domains, respectively) were constructed. Co-immunoprecipitation analysis confirmed successful construction as both Flag-tagged TBC1D15 (WT) and TBC1D15 (R400K) were able to bind with mitochondrial Fis1 while Flag-tagged TBC1D15 (Δ231-240) failed to bind Fis1 (Figure 5B). Meanwhile, western blot analysis showed that Fis1 and RAB7 protein levels were elevated after 3-day MI while TBC1D15 failed to alter both protein changes induced by 3-day MI (Figure S6A-B), indicating that acute MI-induced Fis1/RAB7 protein changes could facilitate the downstream effects of TBC1D15. Previous study has reported that the number of degradation enzymes in lysosomes remains unchanged when exposed to hypoxia 48. Therefore, we focused on the activity of lysosomal enzymes by detecting the lysosomal acidification, essential for lysosomal enzymes degradation activity. Lyso-Sensor Green DND-189 staining, indicative of lysosomal acidification, revealed dramatically decreased acidic fluorescence (22.6 ± 2.8 *vs.* 181.2 ± 16.3 from Normoxia, *P* < 0.05) and increased lysosomal size (2.220 ± 0.201 *vs.* 0.135 ± 0.011 from Normoxia, *P* < 0.05) under 9-h hypoxia. TBC1D15 (WT) improved lysosomal acidification (86.5 ± 5.5 *vs.* 22.6 ± 2.8 from Hypoxia, *P* < 0.05) and lysosomal size (1.143 ± 0.109 *vs.* 2.220 ± 0.201 from Hypoxia, *P* < 0.05) while TBC1D15 (R400K and Δ231-240 mutants) failed to exert improvement induced by TBC1D15 (WT) (Figure 5C-E). To further examine the degradation of LC3-conjugated cargoes by lysosomes, immunofluorescence of LC3 and LAMP1 was employed. Results showed that LC3-positive “granules” (green), indicator of cargoes for degradation, were enlarged and accumulated in 9-h hypoxic cardiomyocytes. LC3-positive “granules” encompassed by enlarged LAMP1-positive structures (red), indicator of autolysosomes, were also accumulated in cardiomyocytes under 9-h hypoxic stress (18.20 ± 1.53 *vs.* 1.00 ± 0.39 from Normoxia, *P* < 0.05), in line with LAMP1 and COXIV immunofluorescence. With TBC1D15 (WT) overexpression, the LC3-positive “granules” and LAMP1 encompassed LC3-positive “granules” were drastically decreased (8.70 ± 0.68 *vs.* 18.20 ± 1.53 from Hypoxia, *P* < 0.05), the effects of which were reversed by TBC1D15 (R400K and Δ231-240 mutants) (Figure 5F-G). These findings suggested that Fis1 binding and RAB7-GAP domains are involved in TBC1D15-dependent lysosomal size and acidification restoration, and subsequent mitophagy regulation.

### Lysosomal acidification restoration may be associated with TBC1D15/Fis1/RAB7-dependent mitochondria-lysosome contacts untethering

**Figure 6:**
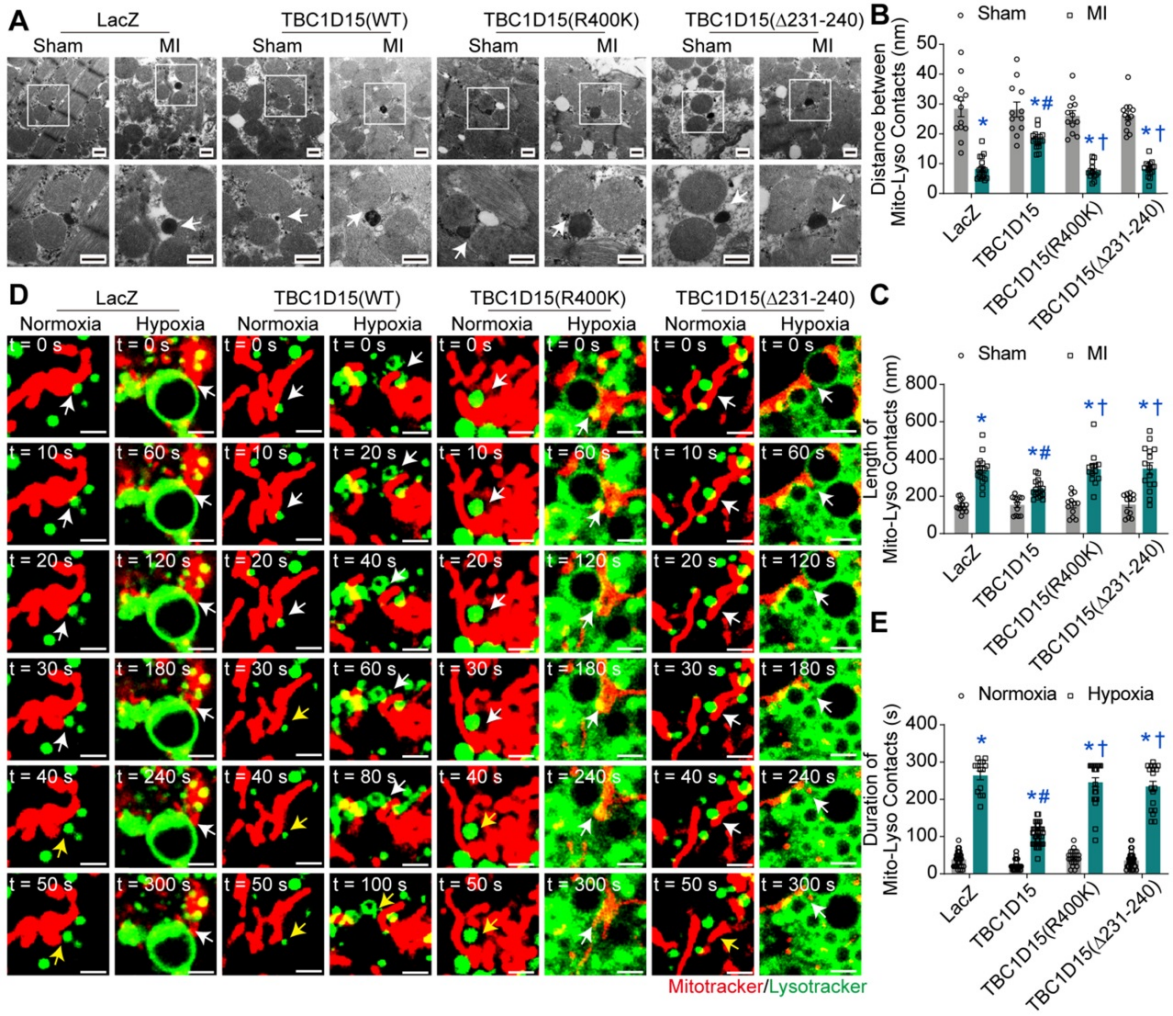
** TBC1D15 regulates mitochondria-lysosome contacts dependent on its Fis1 binding and RAB7-GAP domains. A-C.** Mice were transfected with LacZ or TBC1D15 adenoviruses (WT, R400K or Δ231-240) in the absence or presence of 3-day MI. MI-induced decrease of distance and increase of length of mitochondria-lysosome contacts were attenuated by TBC1D15 overexpression, but not by mutant TBC1D15 (R400K) or TBC1D15 (Δ231-240) (n = 12-18). Representative TEM images of mitochondria-lysosome contacts (Scale bar = 500 nm) are shown. Rectangles exhibit magnified views. The white arrows indicate the representative mitochondria-lysosome contacts. Mean ± SEM, * *p* < 0.05 *vs.* corresponding Sham group; # *p* < 0.05 *vs.* MI-LacZ group; † *p* < 0.05 *vs.* MI-TBC1D15 group; **D-E.** NMCMs were transfected with LacZ or TBC1D15 adenoviruses (WT, R400K or Δ231-240) in the absence or presence of 9-h hypoxia. Hypoxia-induced increase of duration of mitochondria-lysosome contacts was ameliorated by TBC1D15 overexpression, but not by mutant TBC1D15 (R400K) or TBC1D15 (Δ231-240) (n = 14-42). Representative confocal live cell time-lapse images of mitochondria-lysosome contacts (white arrows) and contacts untethering (yellow arrows) (Scale bar = 2 µm) are displayed. Mean ± SEM, * *p* < 0.05 *vs.* corresponding Normoxia group; # *p* < 0.05 *vs.* Hypoxia-LacZ group; † *p* < 0.05 *vs.* Hypoxia-TBC1D15 group.

Both Fis1 binding and RAB7-GAP domains are involved in mitochondria-lysosome contacts, critical for both mitochondrial and lysosomal dynamics 29. To this end, we examined the mitochondria-lysosome contacts in cardiomyocytes following acute MI or long-term hypoxia using TEM and confocal live time-lapse imaging. Static results from TEM imaging revealed that the distance of mitochondria-lysosome contacts was decreased (8.23 ± 0.86 *vs.* 28.43 ± 2.65 from Sham, *P* < 0.05) while the length of mitochondria-lysosome contacts was increased (341.8 ± 17.1 *vs.* 151.3 ± 9.7 from Sham, *P* < 0.05) in peri-infarct myocardium 3 days after MI, indicating elevated tight mitochondria-lysosome contacts following acute MI. TBC1D15 (WT) increased distance (18.13 ± 0.72 *vs.* 8.23 ± 0.86 from MI, *P* < 0.05) and decreased length (239.8 ± 11.1 *vs.* 341.8 ± 17.1 from MI, *P* < 0.05) of mitochondria-lysosome contacts while TBC1D15 (R400K and Δ231-240 mutants) failed to attenuate the elevation of mitochondria-lysosome contacts induced by 3-day MI (Figure 6A-C). Dynamic observation from confocal live cell time-lapse imaging revealed that mitochondria (Mito-Tracker, red) tended to be in contact with extremely enlarged lysosomes (Lyso-Tracker, green) for an excessively prolonged duration under 9-h hypoxic condition (264.3 ± 11.8 *vs.* 38.7 ± 3.7 from Normoxia, *P* < 0.05), and even after belated contacts release, these enlarged lysosomes were unlikely to timely recover to pre-hypoxia levels, the effect of which was abrogated by TBC1D15 (WT) (110.0 ± 4.7 *vs.* 264.3 ± 11.8 from Hypoxia, *P* < 0.05) while not by TBC1D15 (R400K and Δ231-240 mutants) (Figure 6D-E and Video S1-8). In light of previous results, these findings depicted an essential role for mitochondria-lysosome contacts in TBC1D15-mediated lysosomal regulation and mitophagy flux activation.

### Fis1 binding and RAB7-GAP domains are indispensable for TBC1D15-dependent cardioprotective effects

**Figure 7:**
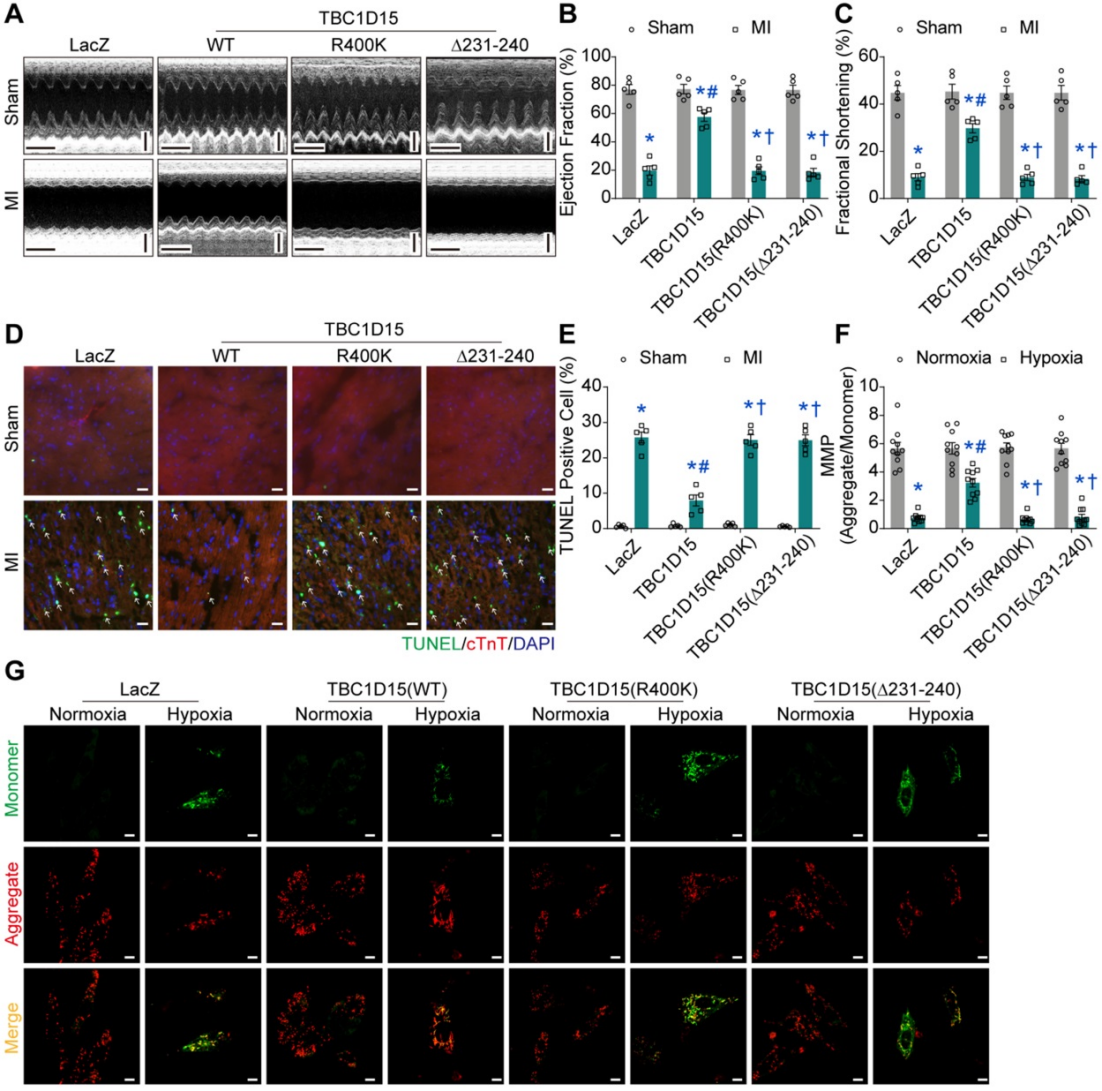
** Fis1 binding and RAB7-GAP domains are indispensable for TBC1D15-dependent cardioprotective effects. A-C.** Decreases of ejection fraction and fractional shortening after 3-day MI were attenuated by TBC1D15 overexpression, but not by mutant TBC1D15 (R400K) or TBC1D15 (Δ231-240) (n = 5). Representative echocardiographic images (Scale bar = 200 ms-horizontal and 2 mm-vertical) are displayed; **D-E.** Increase of myocardial apoptosis after 3-day MI was alleviated by TBC1D15 overexpression, but not by mutant TBC1D15 (R400K) or TBC1D15 (Δ231-240) (n = 5). Representative TUNEL/cTnT/DAPI staining images (Scale bar = 25 µm) are shown. The white arrows indicate TUNEL positive nuclei. Mean ± SEM, * *p* < 0.05 *vs.* corresponding Sham group; # *p* < 0.05 *vs.* MI-LacZ group; † *p* < 0.05 *vs.* MI-TBC1D15 group; **F-G.** Cardiomyocytes mitochondrial membrane potential (MMP) loss following 9-h hypoxia was ameliorated by TBC1D15 overexpression, but not by mutant TBC1D15 (R400K) or TBC1D15 (Δ231-240) (n = 10). Representative images of JC-1 staining (Scale bar = 10 µm) are exhibited. Mean ± SEM, * *p* < 0.05 *vs.* corresponding Normoxia group; # *p* < 0.05 *vs.* Hypoxia-LacZ group; † *p* < 0.05 *vs.* Hypoxia-TBC1D15 group.

To further examine the role of Fis1 binding and RAB7-GAP domains in TBC1D15-regulated cardiac protective effects against ischemia, echocardiography, Evans blue/TTC staining, Masson Trichrome staining and TUNEL staining were performed. Data from echocardiographic assessment showed that TBC1D15 attenuated 3-day MI-induced decrease in ejection fraction (57.53 ± 3.05 *vs.* 19.77 ± 3.27 from MI, *P* < 0.05) and fractional shortening (29.78 ± 1.97 *vs.* 8.99 ± 1.56 from MI, *P* < 0.05) as described in Figure 1. Interestingly, both TBC1D15 (R400K and Δ231-240) mutants failed to recapitulate wild-type TBC1D15-elicited benefits against 3-day MI-induced cardiac dysfunction (Figure 7A-C). Along the same line, TBC1D15 attenuated the increase of LVESD (2.602 ± 0.157 *vs.* 4.188 ± 0.173 from MI, *P* < 0.05), LVEDD (3.698 ± 0.170 *vs.* 4.597 ± 0.135 from MI, *P* < 0.05) and LVESV (25.26 ± 3.45 *vs.* 78.86 ± 7.52 from MI, *P* < 0.05) following 3-day MI procedure, the effect of which was canceled by both TBC1D15 mutants (Figure S7A-C). Neither wild-type TBC1D15 nor TBC1D15 mutants alone exerted any effects on EF, FS, LVESD, LVEDD and LVESV. Data from Evans blue/TTC staining showed that under the same degrees of AAR, TBC1D15 attenuated myocardial infarct size following 3-day MI (24.44 ± 1.99 *vs.* 44.52 ± 3.88 from MI, *P* < 0.05), the effect of which was nullified by either TBC1D15 mutant (Figure S7D-F). Data from Masson Trichrome staining showed that TBC1D15 attenuated myocardial interstitial fibrosis resulted from the 3-day MI process (24.47 ± 2.05 *vs.* 45.11 ± 2.59 from MI, *P* < 0.05), which was absent by both TBC1D15 mutants (Figure S7G-H). Furthermore, TUNEL staining showed that both TBC1D15 mutants failed to exert beneficial effect mediated by wild-type TBC1D15 on myocardial apoptosis following 3-day MI procedure (7.96 ± 1.55 *vs.* 25.77 ± 1.54 from MI, *P* < 0.05, Figure 7D-E). Meanwhile, for *in vitro* study, JC-1, ROS and TUNEL staining were performed. Data from JC-1 staining indicated that TBC1D15 attenuated the loss of MMP resulted from 9-h hypoxia (3.244 ± 0.303 *vs.* 0.758 ± 0.097 from Hypoxia, *P* < 0.05) while both TBC1D15 (R400K and Δ231-240) mutants failed to mimic wild-type TBC1D15-dependent benefits against hypoxia-induced reduction of MMP (Figure 7F-G). In addition, ROS detection showed that TBC1D15 alleviated the accumulation of ROS induced by 9-h hypoxia (6.24 ± 0.52 *vs.* 14.56 ± 0.52 from Hypoxia, *P* < 0.05), the effect of which was nullified by both TBC1D15 mutants (Figure S8A-B). TUNEL staining showed that both TBC1D15 mutants failed to exert reduction effect mediated by wild-type TBC1D15 on cardiomyocyte apoptosis following 9-h hypoxia (5.04 ± 0.60 *vs.* 15.13 ± 0.80 from Hypoxia, *P* < 0.05, Figure S8C-D). These findings favored an obligatory role of both functional domains, coding for Fis1 binding and RAB7 GTP hydrolysis, in TBC1D15-dependent cardioprotection.

## Discussion

**Figure 8:**
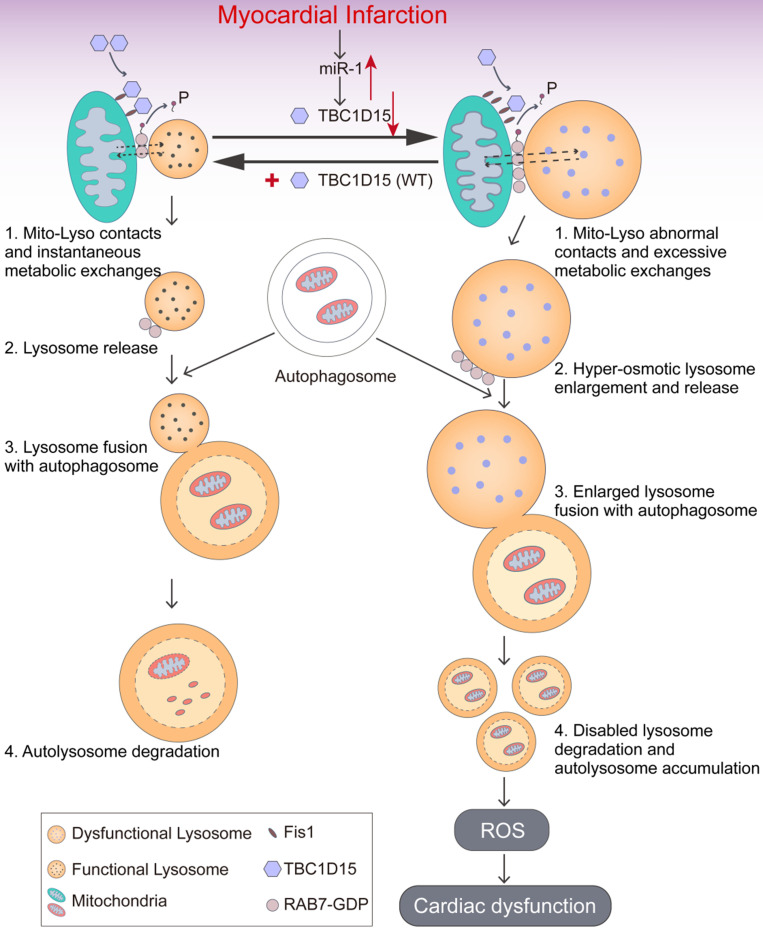
** Working model of TBC1D15-induced cardioprotection in acute MI.** Under ischemia or hypoxic stress, TBC1D15 is downregulated as a result of TBC1D15 mRNA degradation driven by miR-1, leading to compromised recruitment of TBC1D15 to mitochondria via binding with Fis1 and delayed release of lysosomes from mitochondria by RAB7 GTP hydrolysis at the mitochondria-lysosome contact sites. The abnormal mitochondria-lysosome contacts may prolong excessive metabolic exchanges between the two organelles, and elevate lysosomal osmotic pressure, resulting in enlargement, poor acidification and dysfunction of lysosomes, ultimately blockade of lysosome-dependent degradation of damaged mitochondria in ischemia or long-term hypoxia. Accumulation of damaged mitochondria results in loss of mitochondrial membrane potential, excessive ROS production, cardiomyocyte apoptosis, and cardiac dysfunction. Overexpression of TBC1D15 loosens tethering of mitochondria-lysosome contacts, restores lysosome size and acidification, and promotes the ability of lysosome-dependent digestion of depolarized mitochondria.

The salient findings from our study depicted that TBC1D15 preserved cardiac function, decreased infarct area, myocardial fibrosis and cardiomyocyte apoptosis following acute MI. TBC1D15 levels were dramatically downregulated due to elevation of miR-1 in response to acute MI, leading to RAB7-mediated accumulation of abnormal mitochondria-lysosome contacts and subsequently contacts-induced enlargement of lysosomes. The enlarged lysosomes could fuse with autophagosomes to form autolysosomes although cargos inside failed to be degraded within the autolysosomes due to defective acidification. As a result, dysfunctional autolysosomes and ROS accumulation ultimately resulted in cardiac dysfunction. However, TBC1D15 overexpression could translocate onto mitochondria through binding with mitochondrial Fis1 and promote untethering of mitochondria-lysosome contacts via activation of RAB7 GTP hydrolysis, thus preventing enlargement of lysosomes and restoring lysosomal degradation. In consequence, TBC1D15 is capable of promoting clearance of damaged mitochondria for quality control, resulting in preserved myocardial mitochondrial function, mitochondrial integrity and cardiac contractile function in the face of acute MI challenge, as summarized in our scheme (Figure 8). To the best of our knowledge, this is the first study to investigate the role of TBC1D15 in the heart and describe the relationship between mitochondria-lysosome contacts and mitophagy flux in long-term ischemic/hypoxic settings.

The key finding in our study was that TBC1D15 functioned as the critical bridge molecule between mitochondria-lysosome contacts and mitophagy. As a member of TBC-domain-containing protein family, TBC1D15 is known to participate in multiple cellular processes 29, 49-57 and neurological disorders 58. Collectively, TBC1D15 is involved in mitochondrial fission, autophagosome modification, mitochondria-lysosome contacts and transport of cellular substance via endosomal system. However, little is known about the role of TBC1D15 in the heart. In our hands, TBC1D15 level was reduced in ischemic heart while overexpression of TBC1D15 preserved cardiac systolic function and improved myocardial morphology following acute MI, similar with its role in neurological disorders 58. Ample evidence has revealed the deleterious role of mitochondrial damage in the onset and progression of acute MI 7, which correlates well with our results. Furthermore, our study also proved that TBC1D15 exerted protective effects on acute MI-induced mitochondrial abnormalities and ROS accumulation, similar with reported actions of TBC1D15 on mitochondrial morphology 29, 54. Mitochondria in adult cardiomyocytes can be categorized into 3 distinct populations, peri-nuclear, interfibrillar, and subsarcolemmal mitochondria, with geographical distinction in the regulation of mitochondrial metabolism 59. Our data showed no difference in the relative proportion of these 3 mitochondrial populations in all experimental groups. In addition, our results also revealed lack of effect for TBC1D15 on total number of mitochondria and PGC1α levels. These findings have indicated a beneficial role of TBC1D15 on mitochondria possibly through regulation of quality control instead of mitochondrial biogenesis and distribution. Seminal findings from Ong and colleagues depicted the importance of inhibiting excessive mitochondrial fragmentation in conferring cardioprotection against I/R injury 60-62. Either excessive large or small content of mitochondria can be detrimental for cell homeostasis. Although our findings have shed some lights towards a role for TBC1D15 in mitochondrial morphology, further study is still warranted to fully elucidate the role of TBC1D15 in the governance of mitochondrial homeostasis in particular mitochondrial dynamics.

Mitophagy is a highly conserved lysosome-dependent process through which damaged mitochondria are degraded and recycled for mitochondrial quality control. Dysregulation of mitophagy participates in the pathophysiology of cardiovascular disease 26. Autophagy has been examined in MI-induced myocardial abnormalities but whether autophagy activity is upregulated or downregulated during the different phases of MI has remained a question of debate 63. Here, our findings revealed that following coronary artery ligation, level of LC3II was initially increased, but then returned to a sub-normal level. On the other hand, p62 level declined 2 h following infarction, and then raised beyond normal level at 72 h post-infarction. These findings indicate activation of autophagy during early acute phase of MI followed by dampened autophagy in late acute period. Further assessment of autophagy or mitophagy flux using mRFP-GFP-LC3, mito-Keima and bafilomycin A1 treatment revealed that long-term hypoxia induced accumulation of autolysosomes instead of autophagosomes, indicating the blockage of autophagy flux, similar with the finding of impaired autophagosomes clearance in myocardial I/R injury 48. Accumulation of autolysosomes depends on decreased efficiency of degradation and/or increased rate of uptake (fusion). In fact, the decrease in autophagosomes or LC3II level may indirectly signify a decreased rate of uptake while lysosomal dysfunction or p62 level may indirectly reveal the decreased efficiency of digestion. However, the exact rate of uptake or efficiency of digestion needs more comprehensive dynamic investigation. Both immunofluorescence of COXIV or LC3 with LAMP1 and Lyso-Sensor detection confirmed the existence of lysosomes enlargement with disabled acidification following long-term hypoxia. However, the activity of autophagy may be a little different from previous reports as different phases investigated, similar with the tendency of autophagy activity in transverse aortic constriction (TAC) mouse model 30. Overexpression of TBC1D15 effectively dampened number of enlarged lysosomes, restored lysosomal acidification and activated mitophagy flux, coinciding with TBC1D15-regulated lysosomal morphology 55 and lysosomal activation in autophagy flux regulation 41.

Recent findings have proved the functional interaction between mitochondria and lysosomes in a neuropathological context 64. To date, different pathways of interaction between mitochondria and lysosomes have been described including mitophagy, transfer of mitochondria-derived vesicles (MDVs) and mitochondria-derived compartments (MDCs), and direct physical membrane contact between mitochondria and lysosomes 28. A more recent study denoted that TBC1D15 untethers mitochondria-lysosome contacts through binding with Fis1 and activating RAB7 GTP hydrolysis, and subsequently regulates mitochondrial and lysosomal morphology 65. Here, we demonstrated that loss of TBC1D15 (as in the case of acute MI)-induced abnormal mitochondria-lysosome contacts were observed in both peri-infarct myocardium and long-term hypoxic cardiomyocytes which was ameliorated by TBC1D15 re-expression. Furthermore, either TBC1D15 mutant lacking Fis1 binding or RAB7 GAPase activation failed to modulate lysosomal acidification, mitophagy flux activation and cardioprotective effects mediated by TBC1D15, indicating an essential role of mitochondria-lysosome contacts in acute MI-induced lysosomal dysregulation, mitophagy flux blockage and cardiac abnormalities. In this way, this work also provides the possibility that TBC1D15 promotes the shift between different pathways of mitochondria and lysosome interaction (from mitochondria-lysosome contacts to mitophagy). Mitochondria-lysosome contacts may function as platforms for metabolic exchanges between the two organelles, as inter-organelle communication is usually mediated by membrane contact sites 66, 67. Dysregulation of these metabolic exchange processes may lead to excessive accumulation of substance, enlargement of lysosomes and subsequent lysosomal dysfunction. However, more work would be taken to explore the specific mechanism(s) involved in RAB7-mediated mitochondria-lysosome contacts and lysosomal enlargement.

In summary, TBC1D15 exerts protective effects on the function and morphology of infarct heart through untethering mitochondria-lysosome contacts via Fis1/RAB7 pathway and subsequently keeping good mitochondrial quality control by restoring lysosomal acidification. This work may provide a new target in the clinical treatment of acute MI. However, more in-depth scrutiny should be engaged towards understanding the role of TBC1D15 in other pathological conditions especially for cardiovascular diseases.

## Abbreviations

MI: myocardial infarction
ATP: adenosine triphosphate
DAPI: 4',6-diamidino-2-phenylindole
DMEM: Dulbecco's Modified Eagle Medium
EF: ejection fraction
FBS: fetal bovine serum
FCCP: carbonyl cyanide 4-(trifluoromethoxy)phenylhydrazone
Fis1: Fission 1
FS: fractional shortening
GAP: GTPase-activating protein
GDP: guanosine diphosphate
GTP: guanosine triphosphate
HR: heart rate
LAD: left anterior descending branch
LC3: light chain 3
LVEDD: left ventricular end diastolic diameter
LVESD: left ventricular end systolic diameter
LVESV: left ventricular end systolic volume
MDCs: mitochondria-derived compartments
MDVs: mitochondria-derived vesicles
MI: myocardial infarction
MMP: mitochondrial transmembrane potential
NMCMs: neonatal mouse cardiomyocytes
OCR: Oxygen consumption rate
PINK1: PTEN-induced putative kinase-1
ROS: reactive oxygen species
SQSTM1/p62: sequestosome-1/p62
TAC: transverse aortic constriction
TBC: Tre2/Bub2/Cdc16
TBC1D15: TBC1 domain family, member 15
TEM: transmission electron microscope
TTC: 5-triphenyltetrazolium chloride
TUNEL: TdT-mediated dUTP nick-end labeling
VDAC: voltage-dependent anion channel
WT: wild-type

## Supplementary Material

Supplementary figures and tables.

Supplementary vedios/movies.
