# Supplementary material for: TBC1D15/RAB7-regulated mitochondria-lysosome interaction confers cardioprotection against acute myocardial infarction-induced cardiac injury
Source: Theranostics. 2020 Sep 14;10(24):11244–63. doi: 10.7150/thno.46883 (PMC7532681; doi:10.7150/thno.46883)
Supplement: Supplementary file 1 — Supplementary figures and tables. [file thnov10p11244s1.pdf]

Supplemental Materials

TBC1D15/RAB7-regulated mitochondria-lysosome interaction confers cardioprotection against acute myocardial infarction-induced cardiac injury

Wenjun Yu[1*], Shiqun Sun[1*], Haixia Xu[1,2*], Congye Li[3], Jun Ren[1,4] and Yingmei Zhang[1#]

1 Department of Cardiology and Shanghai Institute of Cardiovascular Diseases, Zhongshan Hospital, Fudan University, Shanghai 200032, China.

2 Department of Cardiology, Affiliated Hospital of Nantong University, Jiangsu 226001, China.

3 Department of Cardiology, Xijing Hospital, Air Force Medical University, Xi'an, 710032, China.

4 Center for Cardiovascular Research and Alternative Medicine, University of Wyoming, Laramie, WY 82071, USA.

Running title: TBC1D15 preserves cardiac function under acute MI

* Wenjun Yu, Shiqun Sun and Haixia Xu contributed equally to this work

# Correspondence author:

Yingmei Zhang, MD, PhD, FACC

Department of Cardiology and Shanghai Institute of Cardiovascular Diseases, Zhongshan Hospital Fudan University, 180 Fenglin Road, Shanghai 200032, China.

Tel/Fax: 021-64041990/ 021-64223006

E-mail: zhangym197951@126.com.

Figure S1

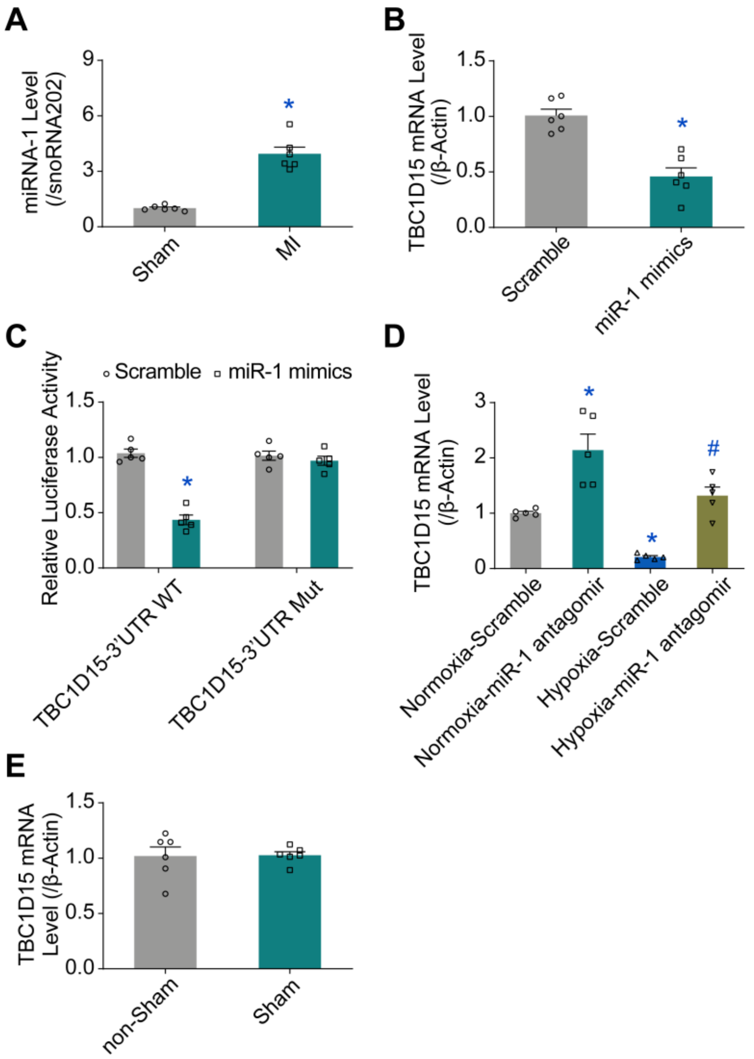

Figure S1: Upregulation of miR-1-reduced TBC1D15 mRNA level following acute MI. A. Level of miR-1 was upregulated in response to a 3-day MI procedure, n = 6, Mean ± SEM, * $p < 0.05$ *vs.* Sham group; **B.** TBC1D15 mRNA level was downregulated in NMCMs treated with the miR-1 mimics, n = 6, Mean ± SEM, * $p < 0.05$ *vs.* Scramble group; **C.** TBC1D15 luciferase activity driven by the miR-1 mimics was decreased in NMCMs treated with wild-type, but not mutant TBC1D15-3'UTR, n = 5, Mean ± SEM, * $p < 0.05$ *vs.* Scramble group; **D.** TBC1D15 mRNA level was downregulated in hypoxic NMCMs while it was upregulated by the miR-1 antagomir in the absence or presence of a 9-h hypoxia challenge, n = 5, Mean ± SEM, * $p < 0.05$ *vs.* Normoxia-Scramble group; # $p < 0.05$ *vs.* Hypoxia-Scramble group; **E.** Effect of sham operation on TBC1D15 mRNA level, n = 6, Mean ± SEM.

Figure S2

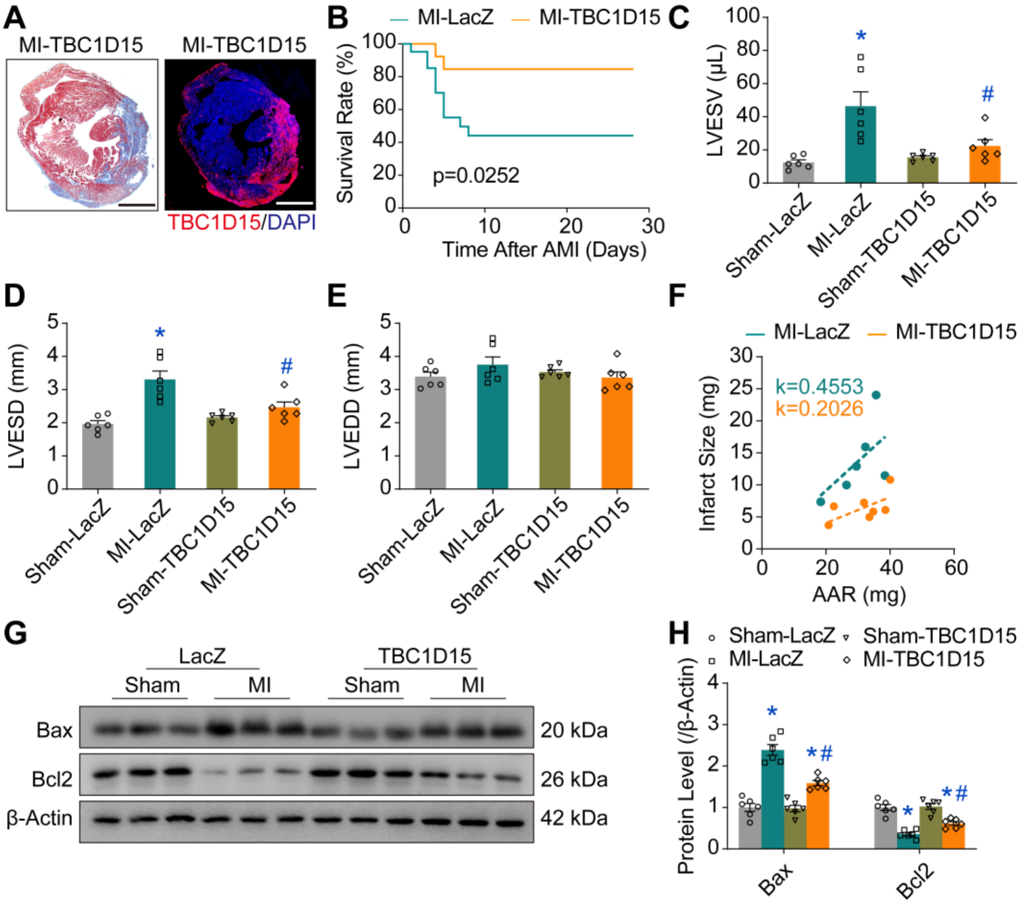

Figure S2: Protection of TBC1D15 overexpression against cardiac injury following acute MI. Mice were transfected with LacZ and TBC1D15 adenovirus in the absence or presence of a 3-day MI challenge. **A.** TBC1D15 expression was validated following myocardial injection of the TBC1D15 adenovirus. Representative histological image of Masson Trichrome staining and corresponsive image of immunofluorescence with DAPI (blue) and TBC1D15 (Red) staining in TBC1D15 overexpressed mice following acute MI challenge (Scale bar = 1 mm) are shown; **B.** Survival rate was increased by TBC1D15 transfection under acute MI (n = 12-14). Kaplan-Meier survival curves are displayed; **C-D.** MI-induced elevations of left ventricular end systolic volume (LVESV) and left ventricular end systolic diameter (LVESD) were alleviated by TBC1D15 overexpression (n = 6); **E.** TBC1D15 exhibited little effect on left ventricular end diastolic diameter (LVEDD) in the absence or presence of acute MI (n = 6); **F.** MI-induced increase of myocardial infarct size/area at risk (AAR) was ameliorated by TBC1D15 overexpression (n = 6); **G-H.** MI-induced increase of Bax level and decrease of Bcl2 level (normalized to β-Actin) were attenuated by TBC1D15 overexpression (n = 6). Mean ± SEM, * $p < 0.05$ *vs.* Sham-LacZ group; # $p < 0.05$ *vs.* MI-LacZ group.

Figure S3

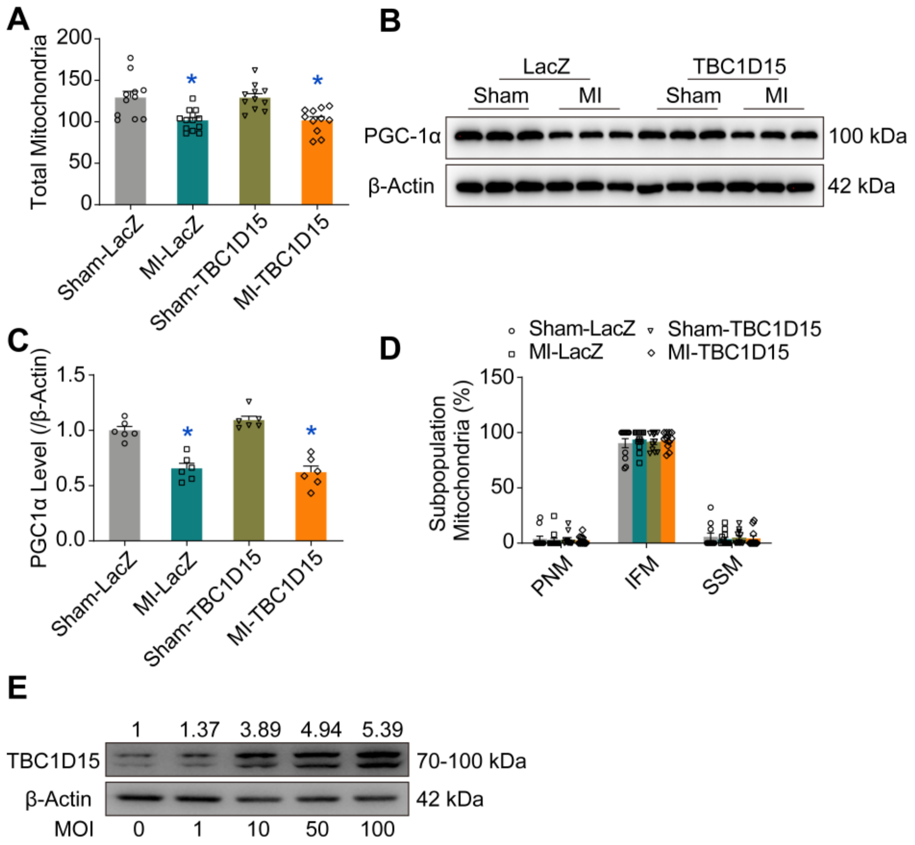

Figure S3: Lack of effect for TBC1D15 on the number and composition of mitochondria. A. Little effect of TBC1D15 on the 3-day MI-induced reduction of total mitochondria number was shown (n = 11-13); **B-C.** Little effect of TBC1D15 on the 3-day MI-induced reduction of PGC1α level (normalized to β-Actin) was displayed (n = 6); **D.** Little effects of TBC1D15 on the composition (distribution) of three mitochondrial population were exhibited (n = 11-13). PNM: peri-nuclear mitochondria; IFM: interfibrillar mitochondria; SSM: subsarcolemmal mitochondria; **E.** Relative TBC1D15 levels (normalized to β-Actin) were illustrated after adenovirus transfection at different MOI (0, 1, 10, 50, 100) in the absence of hypoxia. Mean ± SEM, * $p < 0.05$ *vs.* Sham-LacZ group.

Figure S4

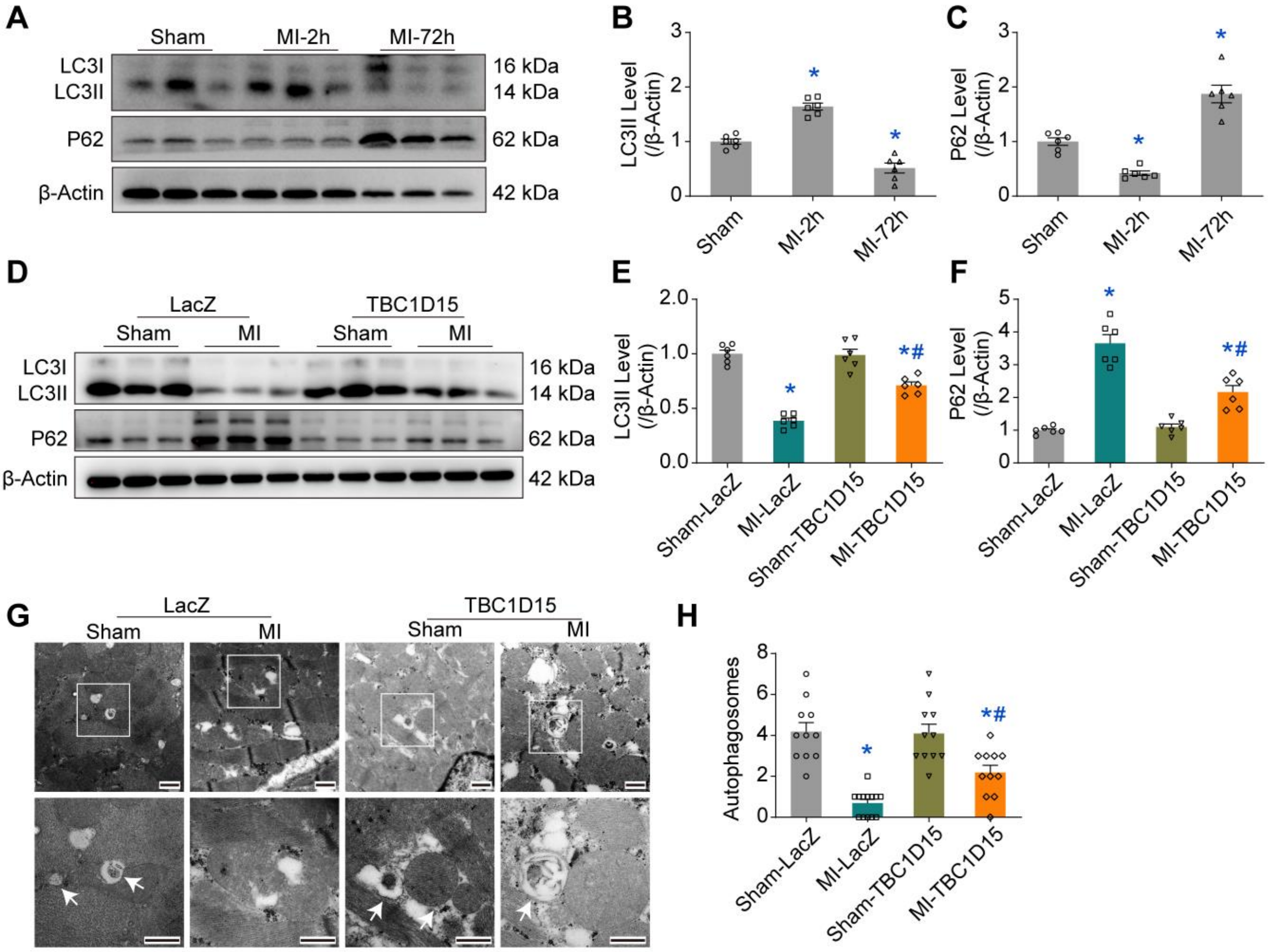

Figure S4: Effect of TBC1D15 on hypoxia-induced suppression of cardiomyocyte autophagy. A-C. Decreased p62 level and increased LC3II level were observed 2 h after MI while increased p62 level and decreased LC3II level were shown 72 h after MI (normalized to β-Actin, n = 6). Mean ± SEM, * $p < 0.05$ *vs.* Sham group; **D-F.** Increase of p62 level and decrease of LC3II level evoked by 72-h MI were attenuated by TBC1D15 overexpression (normalized to β-Actin, n = 6); **G-H.** Decrease of autophagosome number evoked by 72-h MI was alleviated by TBC1D15 (n = 11-13). Representative TEM images of autophagosomes (Scale bar = 500 nm) are shown. Rectangles denote magnified images. The white arrows indicate autophagosomes. Mean ± SEM, * $p < 0.05$ *vs.* Sham-LacZ group; # $p < 0.05$ *vs.* MI-LacZ group.

Figure S5

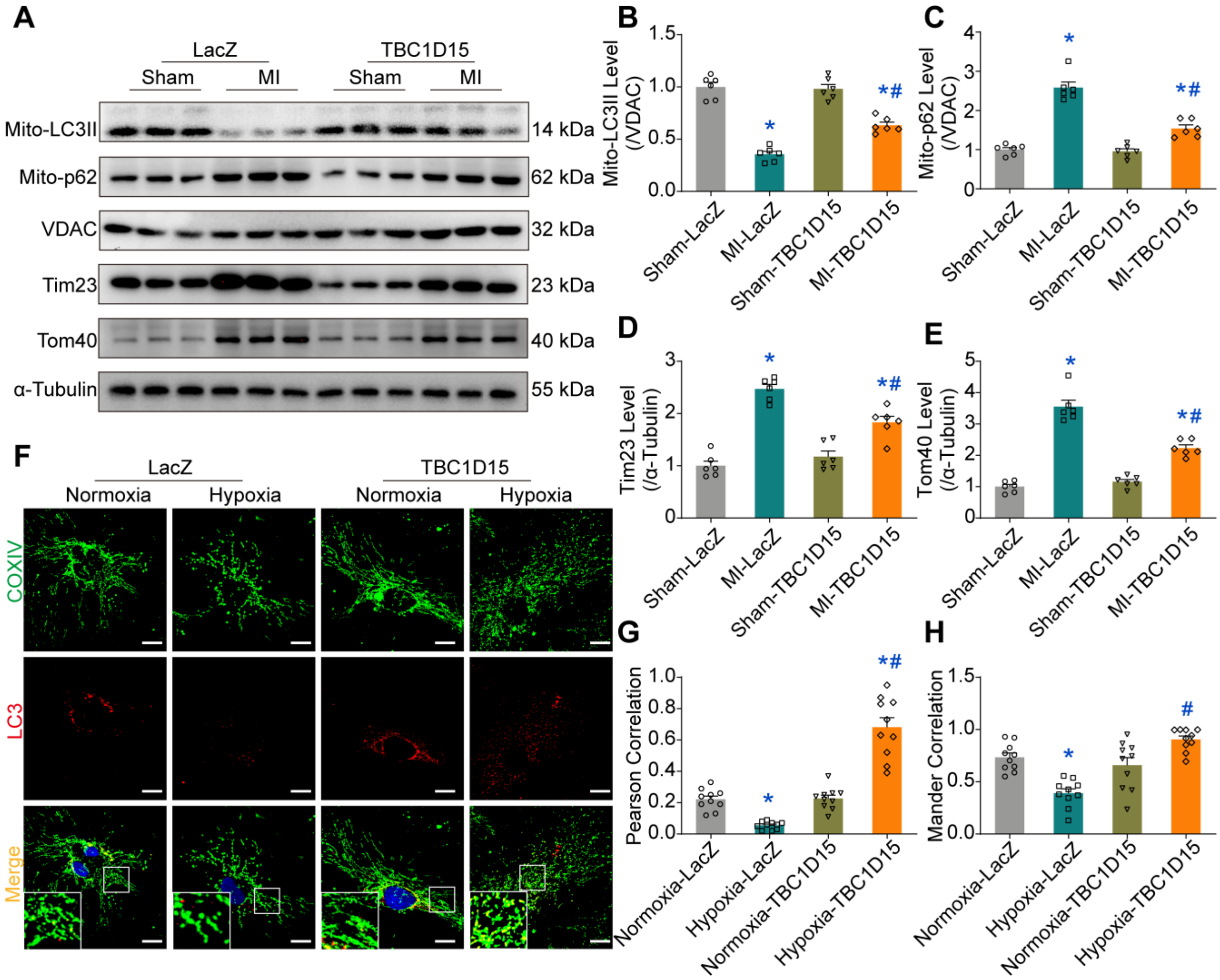

Figure S5: Effect of TBC1D15 on hypoxia-induced suppression of cardiomyocyte mitophagy. A-E. Decrease of mito-LC3II level and increase of protein levels (mito-p62, Tim23 and Tom40) triggered by 72-h MI were attenuated by TBC1D15 (n = 6). Mito-LC3II and mito-p62 levels were normalized to VDAC. Tim23 and Tom40 levels were normalized to α-Tubulin. Mean ± SEM, * $p < 0.05$ *vs.* Sham-LacZ group; # $p < 0.05$ *vs.* MI-LacZ group; **F-H.** Decrease of correlation coefficient (the Pearson correlation and the Mander correlation) of COXIV and LC3 evoked by 9-h hypoxia was alleviated by TBC1D15 (n = 10). Representative immunofluorescence images of COXIV (green) and LC3 (red) (Scale bar = 5 μm) are shown. Rectangles denote magnified views. Mean ± SEM, * $p < 0.05$ *vs.* Normoxia-LacZ group; # $p < 0.05$ *vs.* Hypoxia-LacZ group.

Figure S6

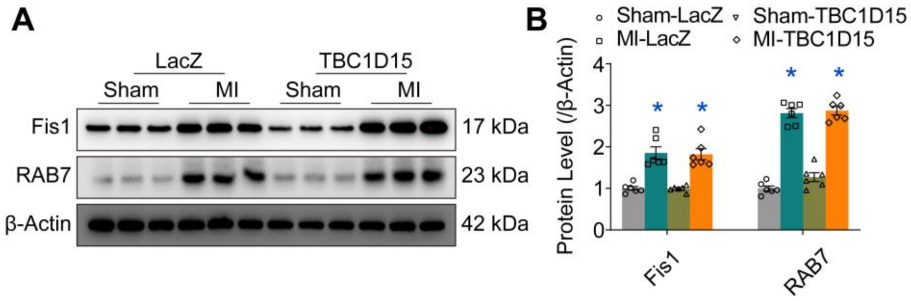

Figure S6: Lack of effect for TBC1D15 on acute MI-upregulated levels of Fis1 and RAB7. A-B. Little effects of TBC1D15 on 72-h MI-induced upregulation of Fis1 and RAB7 levels (normalized to β-Actin) were observed. n = 6, Mean ± SEM, * $p < 0.05$ *vs.* Sham-LacZ group.

Figure S7

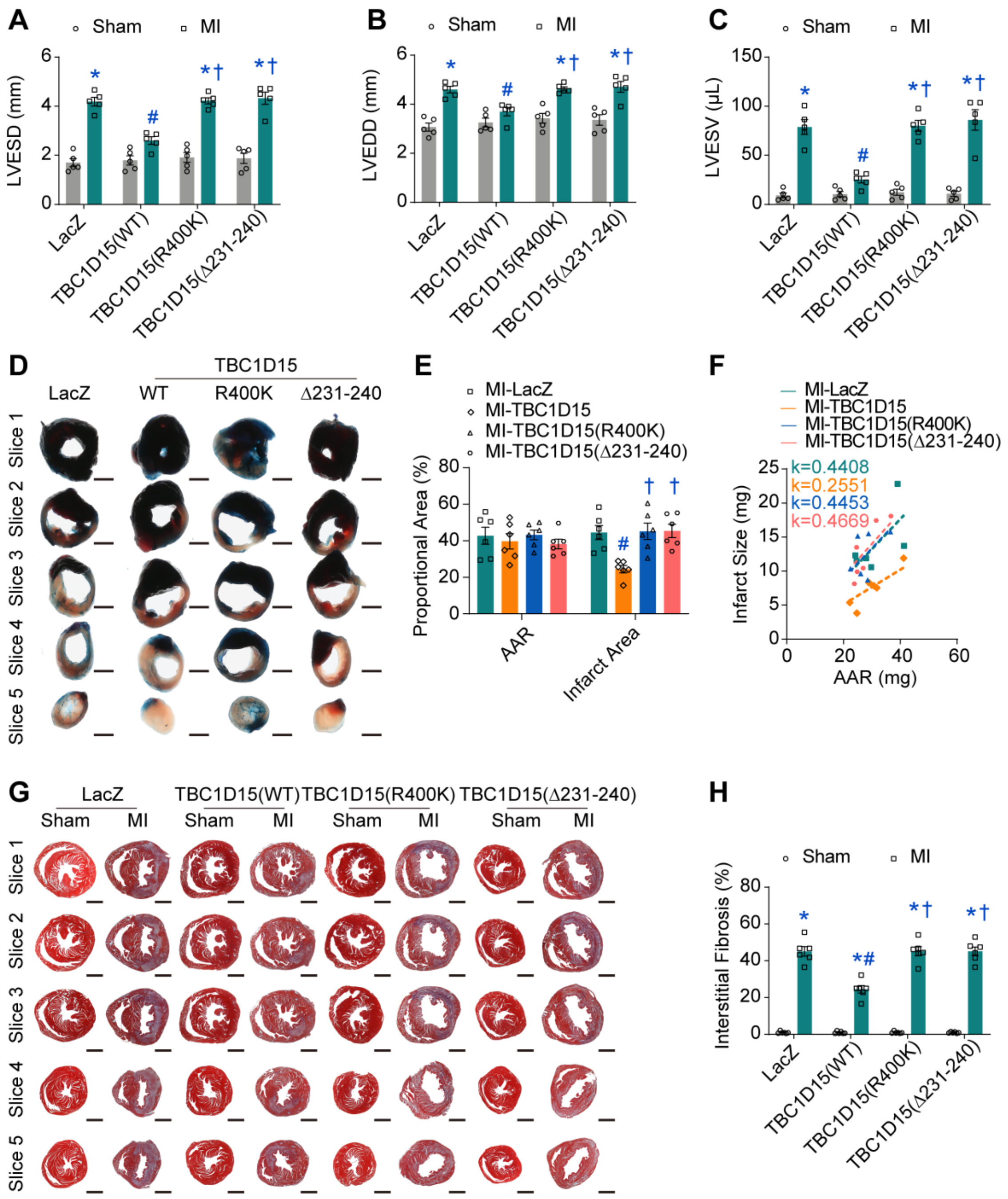

Figure S7: Indispensable role for Fis1 binding and RAB7-GAP domains in TBC1D15-dependent cardioprotective effects. Mice were transfected with LacZ or TBC1D15 adenoviruses (WT, R400K or Δ231-240) in the absence or presence of 3-day MI. **A-C.** MI-induced increases of left ventricular end systolic diameter (LVESD), left ventricular end diastolic diameter (LVEDD) and left ventricular end systolic volume (LVESV) were attenuated by TBC1D15 overexpression, but not by mutant TBC1D15 (R400K) or TBC1D15 (Δ231-240) (n = 5); **D-F.** MI-induced increase of myocardial infarct size/area at risk (AAR) was alleviated by TBC1D15

overexpression, but not by mutant TBC1D15 (R400K) or TBC1D15 (Δ231-240) (n = 6). Representative five-consecutive sections of Evans blue/TTC staining (Scale bar = 1 mm) are shown; **G-H.** MI-induced increase of myocardial interstitial fibrosis was ameliorated by TBC1D15 overexpression, but not by mutant TBC1D15 (R400K) or TBC1D15 (Δ231-240) (n = 6). Representative five-sections of representative Masson Trichrome staining (Scale bar = 1 mm) are displayed. Mean ± SEM, * $p < 0.05$ *vs.* corresponding Sham group; # $p < 0.05$ *vs.* MI-LacZ group; † $p < 0.05$ *vs.* MI-TBC1D15 group.

Figure S8

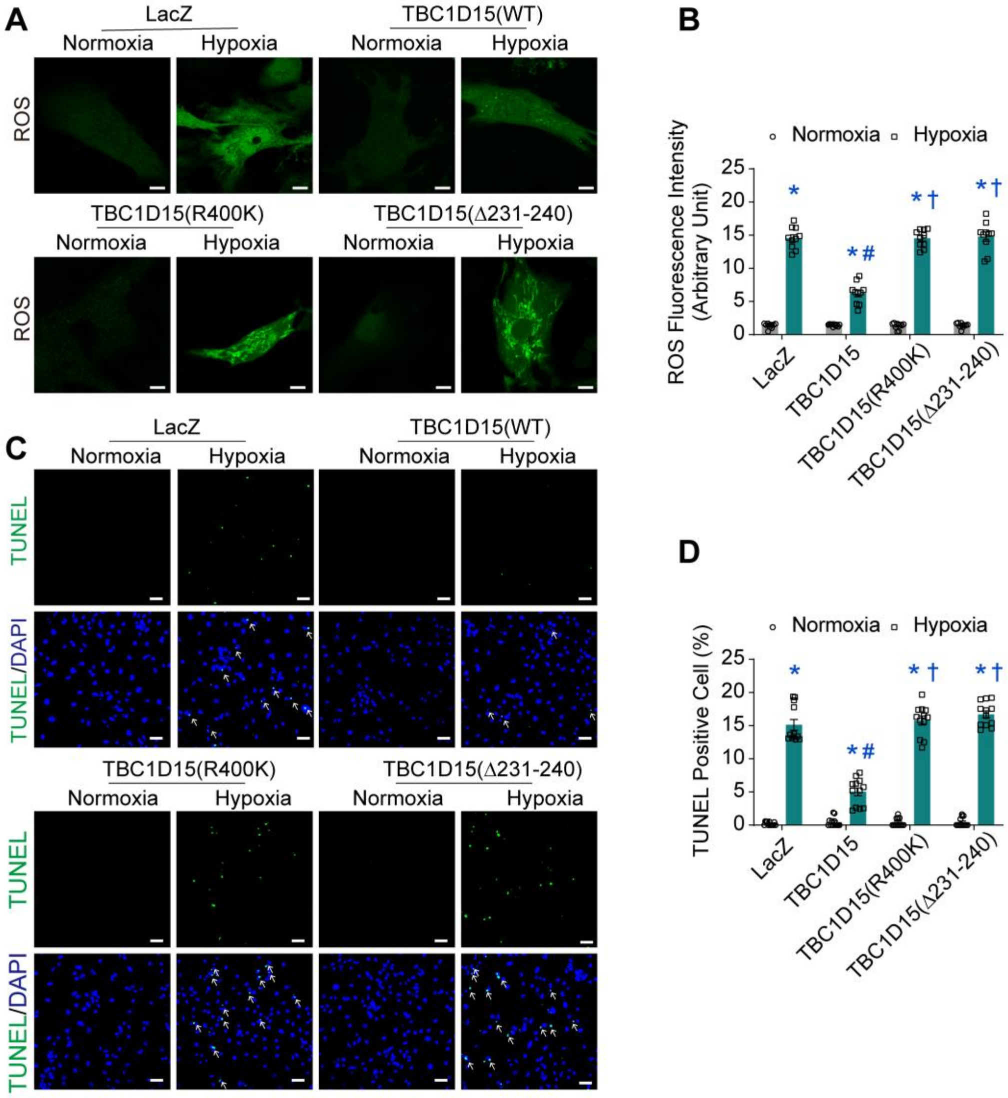

Figure S8: Indispensable role for Fis1 binding and RAB7-GAP domains in TBC1D15-offered cardioprotective effects. NMCMs were transfected with LacZ or TBC1D15 adenoviruses (WT, R400K or Δ231-240) in the absence or presence of a 9-h hypoxia challenge. **A-B.** Hypoxia-induced cardiomyocyte reactive oxygen species (ROS) accumulation was attenuated by TBC1D15 overexpression, but not by mutant TBC1D15 (R400K) or TBC1D15 (Δ231-240) (n = 10). Representative images of DCFH-DA staining (Scale bar = 10 μm) are shown; **C-D.** Hypoxia-induced increase of cardiomyocyte apoptosis was alleviated by TBC1D15 overexpression, but not by mutant TBC1D15 (R400K) or TBC1D15 (Δ231-240) (n = 12). Representative images of TUNEL/DAPI staining (Scale bar = 25 μm) are displayed. The white arrows indicate TUNEL positive nuclei. Mean ± SEM, * $p < 0.05$ *vs.* corresponding Normoxia group; # $p < 0.05$ *vs.* Hypoxia-LacZ group; † p <

0.05 *vs*. Hypoxia-TBC1D15 group.

Video S1-8 Confocal live cell time-lapse imaging of mitochondria-lysosome contacts in NMCMs transfected with LacZ or TBC1D15 adenoviruses (WT, R400K or Δ231-240) treated with or without long-term hypoxia (corresponsive to Figure 6D-E). NMCMs were transfected with LacZ or TBC1D15 adenoviruses (WT, R400K or Δ231-240) at the MOI of 10 for 48 h, and were then exposed to hypoxia for 9 h. To monitor mitochondria and lysosomes, Mito-Tracker Red (mitochondria; red) and Lyso-Tracker Green DND-26 (lysosome; green) were used at the concentrations of 100 nM and 50 nM for the indicated duration.

Video 1-4 In normoxic condition, a lysosome underwent momentary contact with a mitochondron and then quickly untethered from the contact site for dynamic regulation. TBC1D15 (WT), TBC1D15 (R400K) or TBC1D15 (Δ231-240) failed to exert any effects on mitochondria-lysosome contacts in NMCMs under normoxic condition.

Video 5 Long-term hypoxic stress induced longer duration of mitochondria-lysosome contacts, leading to subsequent enlargement of lysosome.

Video 6 TBC1D15 (WT) overexpression significantly shortened the duration of mitochondria-lysosome contacts and restored the morphology of lysosome.

Video 7-8 TBC1D15 (R400K) or TBC1D15 (Δ231-240) overexpression failed to alter the duration of mitochondria-lysosome contacts induced by TBC1D15 (WT).

Table S1: Information of primary antibodies

Primary antibodies	Host	Dilution and supplier	Application	Catalogue No.
TBC1D15	Rabbit	1:500 (1:50 for IF);<br>Abcam, Cambridge, MA	WB, IF	ab121396
Bax	Rabbit	1:1000;<br>Cell Signaling, Danvers, MA	WB	#5023
Bcl-2	Rabbit	1:1000;<br>Cell Signaling, Danvers, MA	WB	#15071
β-actin-HRP	Rabbit	1:5000;<br>Kang Cheng, Shanghai, China	WB	KC-5A08
COXIV	Mouse	1:200;<br>Cell Signaling, Danvers, MA	IF	#11967
cTnT	Mouse	1:500;<br>Abcam, Cambridge, MA	IF	ab8295
Fis1	Rabbit	1:500;<br>Abcam, Cambridge, MA	WB	ab71498
Flag	Rabbit	1:500;<br>Sigma, Burlington, MA	IP; WB	F7425
LAMP1	Rabbit	1:200;<br>Abcam, Cambridge, MA	IF	ab208943
LC3	Rabbit	1:500; (1:200 for IF)<br>Abcam, Cambridge, MA	WB; IF	ab48394
LC3	Mouse	1:200;<br>Cell Signaling, Danvers, MA	IF	#83506
SQSTM1/p62	Rabbit	1:1000;<br>Cell Signaling, Danvers, MA	WB	#5114
RAB5	Rabbit	1:200;<br>Abcam, Cambridge, MA	IF	ab218624
RAB7	Rabbit	1:1000;	WB	ab137029

		Abcam, Cambridge, MA		
RAB11	Rabbit	1:200;<br>Abcam, Cambridge, MA	IF	ab128913
Tim23	Mouse	1:1000;<br>Santa Cruz, USA	WB	sc514463
Tom40	Mouse	1:1000;<br>Santa Cruz, USA	WB	sc365467
α-Tubulin	Mouse	1:1000;<br>Multi Sciences, China	WB	#ab012-040
VDAC	Rabbit	1:1000;<br>Cell Signaling, Danvers, MA	WB	#4661
vinculin	Rabbit	1:500;<br>Abcam, Cambridge, MA	WB	ab129002
PGC1α	Mouse	1:1000;<br>Protein Tech, Chicago, IL	WB	#66369-1

Table S2: Primer sequences used in real-time PCR

**Primary RNA**	**Host**	**Forward**	**Reverse**
TBC1D15	Mouse	CTCATCTTGCGGAAAGGCAAA	TGCATCATCCAATGGTCTCCA
β-Actin	Mouse	GGCTGTATTCCCCTCCATCG	CCAGTTGGTAACAATGCCATGT
